# Overview of *Salvia miltiorrhiza* as a Potential Therapeutic Agent for Various Diseases: An Update on Efficacy and Mechanisms of Action

**DOI:** 10.3390/antiox9090857

**Published:** 2020-09-13

**Authors:** Inyong Jung, Hyerin Kim, Seongcheol Moon, Hyuk Lee, Bonglee Kim

**Affiliations:** 1 College of Korean Medicine, Kyung Hee University, Hoegidong Dongdaemungu, Seoul 05253, Korea; jaugus@khu.ac.kr (I.J.); hellohihr@khu.ac.kr (H.K.); kanyewest@khu.ac.kr (S.M.); well0304@khu.ac.kr (H.L.); 2 Korean Medicine-Based Drug Repositioning Cancer Research Center, College of Korean Medicine, Kyung Hee University, Hoegidong Dongdaemungu, Seoul 05253, Korea

**Keywords:** *Salvia miltiorrhiza* Bunge, dansam, cancer, cardiovascular diseases, liver diseases, nervous system diseases, anti-inflammation, antioxidant

## Abstract

*Salvia miltiorrhiza* Bunge (*S. miltiorrhiza*) is a medicinal herb that has been used for the treatment for various diseases such as cardiovascular and cerebrovascular diseases in East Asia including Korea. Considering its extensive usage as a therapeutic agent for multiple diseases, there is a need to review previous research regarding its therapeutic benefits and their mechanisms. Therefore, we searched PubMed and PubMed Central for articles reporting its therapeutic effects on certain disease groups including cancers, cardiovascular, liver, and nervous system diseases. This review provides an overview of therapeutic benefits and targets of *S. miltiorrhiza*, including inflammation, fibrosis, oxidative stress, and apoptosis. The findings on multi-functional properties of *S. miltiorrhiza* discussed in this article support the efficacy of *S. miltiorrhiza* extract on various diseases, but also call for further research on the multiple mechanisms that mediate its therapeutic effects.

## 1. Salvia miltiorrhiza Bunge

*Salvia miltiorrhiza* Bunge (*S. miltiorrhiza*), also known as dansam in Korean and danshen in Chinese, has been used for the treatment of cardiovascular and cerebrovascular diseases, especially in Asia [1]. It is a deciduous perennial plant which belongs to genus *Salvia* of Lamiaceae family [2]. The active components of *S. miltiorrhiza* are divided into two groups, one of which is water-soluble phenolics including salvianolic acid A (Sal A), salvianolic acid B (Sal B), lithospermic acid, rosmarinic acid, and R-(+)-β-(3,4-dihydroxyphenyl)lactic acid, named danshensu [3] and the other is lipophilic tanshinones including tanshinone I, tanshinone IIA, tanshinone IIB, cryptotanshinone, and dihydrotanshinone I [4]. Salvianolic acids (including Sal A and Sal B), the most abundant compounds from *S. miltiorrhiza*, are known to exhibit diverse biological activities such as antioxidant [5], anti-inflammatory [6], antithrombotic [7], and cardioprotective activities [8,9], while tanshinones show antitumor [10], cardioprotective [11], neuroprotective and analgesic activities [12], and so forth.

Dried roots of *S. miltiorrhiza*, extracted with various solvents, have been reported to have therapeutic effects on a variety of diseases including cancers [13], cardiovascular diseases [14], liver diseases [15], and nervous system diseases [16]. So far, there have been a small number of review articles on *S. miltiorrhiza* extract, most of which focused on its therapeutic use on a certain disease, mainly cardiovascular diseases [17,18] or Alzheimer’s disease [19]. Given its wide usage on many different diseases, there is a need to review studies addressing therapeutic effects and mechanisms of action of *S. miltiorrhiza* in different groups of diseases. Thus, we aim to review the experimental research according to groups of diseases, which will ultimately contribute to a better understanding of its potentials.

## 2. Methods

### 2.1. Search Strategy

Research papers regarding *S. miltiorrhiza* and related diseases were collected from June 2019 to April 2020 using the PubMed and PubMed Central databases (www.ncbi.gov/pubmed). Medical Subject Headings (MeSH) terms such as “Neoplasms”, “Cardiovascular diseases”, “Liver Diseases”, or “Nervous system diseases” and keywords such as “Salvia miltiorrhiza”, “dansam”, “danshen”, “cancer”, “heart”, “liver”, “brain”, “nerve”, “memory”, “Alzheimer’s”, “dementia”, and “extract” were combined to search for relevant articles. To identify articles of interest, all articles retrieved from two databases were manually reviewed and checked for duplication.

### 2.2. Study Selection Criteria

The inclusion criteria were as follows: (1) articles that reported on the efficacy of whole herb extracts of *S. miltiorrhiza* in any dosage form (extract, injection, tablet, pill, etc.), (2) studies where in vitro, in vivo, or ex vivo experiment were performed, (3) original research articles, (4) articles written in English, (5) articles published between January 2014 and December 2019. The exclusion criteria were: (1) review articles, (2) articles on diseases other than cancer, cardiovascular diseases, liver diseases, and nervous system diseases.

### 2.3. Data Extraction

The extracted information included the publication year, the first author’s name, the disease targeted, extractant, cell line/animal model type, dose and duration of extract administered, efficacy, and mechanisms of action (if reported).

## 3. Cancer

Cancer is a major cause of death worldwide. The incidence and mortality of cancers have risen over the past decade [20,21]. They differ between men and women; for example, the age-standardized incidence rates for all cancers combined in 2018 were around 20 percent higher in men than in women (a difference of 36 per 100,000), and mortality rates 50 percent higher in men than in women (a difference of 39.6 per 100,000) [21]. They vary across regions as well; to illustrate, the incidence for adult liver cancers and hepatocellular carcinoma is increasing in the Western countries, while the trends are quite the opposite in Asia with the numbers remaining still high [22]. This global health issue has been addressed with great interest within the scientific and medical community and, as a result, cancer therapeutics such as chemotherapy, immunotherapy, and targeted therapy have continued to improve over the past six decades [23]. However, several studies reported chemotherapy-induced adverse effects, such as neurotoxicity in patients treated with oxaliplatin [24], thalidomide [25], or cytarabine [26]; nausea and vomiting with cisplatin [27] and the AC (doxorubicin/cyclophosphamide) combination [28]; and bone loss with methotrexate [29]. At this point, there is an inevitable need for a therapeutic agent with no or less side-effects, which will help to cope with the global burden of cancer. Results from multiple studies suggest *S. miltiorrhiza* extract should be considered a plausible option.

### Cancer and S. miltiorrhiza

**Figure 1:**
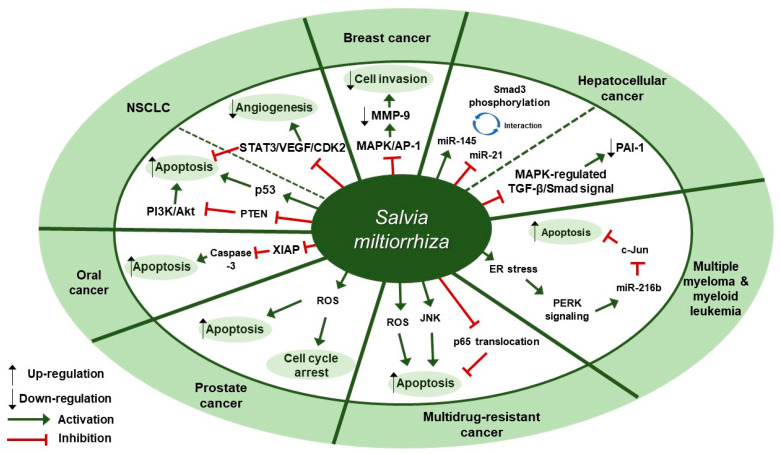
Therapeutic targets of *S. miltiorrhiza* in cancers. NSCLC, non-small cell lung cancer; MAPK, mitogen-activated protein kinase; AP-1, activator protein-1; MMP-9, matrix metalloproteinase-9; miR-145, microRNA-145; miR-21, microRNA-21; PAI-1, plasminogen activator inhibitor 1; ER, endoplasmic reticulum; PERK, protein kinase RNA-like endoplasmic reticulum kinase; miR-216b, microRNA-216b; JNK, c-Jun N-terminal kinase; ROS, reactive oxygen species; XIAP, X-linked inhibitor of apoptosis protein; PTEN, phosphatase and tensin homolog deleted on chromosome ten; PI3K, phosphoinositide 3-kinase; NSCLC, non-small cell lung cancer; STAT3, signal transducer and activator of transcription 3; VEGF, vascular endothelial growth factor; CDK2, cyclin-dependent kinase 2.

Several studies about the anti-cancer effect of *S. miltiorrhiza* have been reported (Table 1). According to Kim et al., treatment with 70% ethanol extract of *S. miltiorrhiza* inhibited matrix metalloproteinase (MMP)-9 expression and cell invasion in 12-O-tetradecanoylphorbol-13-acetate (TPA)-induced MCF-7 breast cancer cells, possibly through down-regulating the mitogen-activated protein kinase (MAPK)/activator protein-1 (AP-1) signaling pathway [30]. Treatment with *S. miltiorrhiza* extract at a dose of 50 µg/mL for 24 h decreased the phosphorylation of MAPKs, including extracellular signal-regulated kinase (ERK), c-Jun N-terminal kinase (JNK), and p38. In addition, it down-regulated phospho (p)-c-Jun expression, implying that *S. miltiorrhiza* extract had an inhibitory effect on TPA-induced MMP-9 expression through blocking the activation of the transcription factor AP-1, a dimer consisting of either Jun/Jun homodimers or Fos/Jun heterodimeric complexes. Wu et al. found that an herbal mixture extract named CASE (Astragalus and Salvia miltiorrhiza water/ethanol extract [71:1.85]) suppressed hepatocellular carcinoma (HCC) progression in vivo (Diethylnitrosamine [DEN]-induced HCC in rats) and in vitro (TGF-β_1_-stimulated HepG2 cells) [31]. At first, 60, 120, or 240 mg/kg of CASE was orally administered to Sprague Dawley (SD) rat with DEN-induced HCC for 28 days, which increased relative microRNA (miR)-145 expression but decreased relative miR-21 expression. In vivo, BALB/c mice xenografted with HepG2 cells were intragastrically injected with 310 mg/kg of CASE once every four days for 28 days, which reversely regulated miR-145 antagomir/miR-21 agomir-mediated Smad3 phosphorylation. CASE up-regulated miR-145 and p-Smad3C expression and down-regulated miR-21, p-Smad3L, p-ERK1/2, p-JNK1/2, and p-p38 expression. According to Boye et al., the same herbal mixture extract (CASE) also affected MAPK-regulated TGF-β/Smad signaling in HCC [32]. In an in vivo mouse model of DEN-induced HCC, administration of CASE at doses of 60, 120, or 240 mg/kg for 12 or 16 weeks suppressed p-ERK, p-JNK, and p-p38 expression. On the other hand, in vivo pretreatment of hepatic stellate cells (HSCs) and/or HepG2 cells with CASE at doses of 20, 40, or 80 µg/mL for 24 h before their stimulation with TGF-β1 decreased p-ERK and p-JNK expression, while it increased p-p38 expression. Pretreatment with CASE also concentration-dependently decreased TGF-β1-induced phosphorylation of oncogenic pSmad3L in HSC and HepG2 cells; reduced nuclear import of Smad4; enhanced the phosphorylation of tumor suppressor pSmad3C especially in HepG2 cells; reduced the expression and nuclear relocation of importins (Imp) 7/8; and suppressed plasminogen activator inhibitor (PAI)-1 gene expression. In Kim et al.’s study, after treatment of U266 (human multiple myeloma) and U937 (human myeloid leukemia) cells with 99.9% ethyl alcohol extract of *S. miltiorrhiza* at doses of 25, 50, 100, or 200 µg/mL for 24 h, cell viability was inhibited in a dose-dependent manner [33]. Further, treatment with *S. miltiorrhiza* extract at doses of 25 or 50 µg/mL for 24 h increased reactive oxygen species (ROS) generation; enhanced phosphorylation of activating transcription factor 4 (ATF4), eukaryotic initiation factor 2 (eIf2), and protein kinase RNA-like endoplasmic reticulum kinase (PERK); and increased CCAAT-enhancer-binding protein homologous protein (CHOP) activation and cleavage of poly ADP-ribose polymerase (PARP) and caspase-3. In addition, 24 h treatment with *S. miltiorrhiza* extract significantly elevated the expression of tumor suppressor miR-216b at a dose of 50 µg/mL, whereas it down-regulated its target protein, c-Jun, at doses of 25 or 50 µg/mL. According to Ye et al., methanol extract of *S. miltiorrhiza* could inhibit the growth of non-small cell lung cancer (NSCLC) via induction of apoptosis through mitochondrial apoptotic pathway and phosphatase and tensin homolog (PTEN)-mediated inhibition of the phosphoinositide 3 kinase (PI3K)/Akt pathway [34]. In vitro treatment with *S. miltiorrhiza* extract at doses of 20 or 40 µg/mL for 24 h induced apoptosis in Glc-82 cells as observed with Annexin V-FITC/PI staining and up-regulated the expression levels of cleaved caspase-3, -9, and PARP1, suggesting the involvement of the mitochondrial apoptotic pathway. In addition, it increased the expression of Bcl-2-associated X protein (Bax) and the tumor-suppressor proteins p53 and p21, while it decreased the expression of B-cell lymphoma 2 (Bcl-2) and B-cell lymphoma-extra large (Bcl-xl), both of which are anti-apoptotic components of Bcl-2 family. Further, treatment with *S. miltiorrhiza* extract also inhibited the phosphorylation of Akt and increased the activity of its upstream inhibitor, PTEN. In vivo, administration of *S. miltiorrhiza* extract at a dose of 40 mg for 22 days suppressed the growth of lung cancer Glc-82 xenografts in Balb/c mice. Lee et al. demonstrated that the arsenic herbal mixture PROS (tetraarsenic hexoxide [PR] + *Olendlandia diffusa* and *Salvia miltiorrhiza* extract (5:2) [OS]) showed apoptotic and anti-angiogenic effects in non-small-cell lung cancer cells (NSCLCs) via inhibition of signal transducer and activator of transcription 3 (STAT3)/vascular endothelial growth factor (VEGF)/cyclin-dependent kinase 2 axis signaling [35]. Treatment with PROS (2.5 µg/mL PR + 180 µg/mL OS) for 24 h exerted significant cytotoxic effects on A549 or H460 better than PR or OS alone and induced apoptosis and S phase arrest, as assessed by DAPI staining and cell cycle analysis. Further, PROS treatment decreased the phosphorylation of STAT3, ERK, proto-oncogene Src, Akt, cyclooxygenase 2 (COX-2), and suppressor of cytokine signaling 1 (SOCS-1) and subsequently inhibited the binding of STAT3 with VEGF or CDK2. PROS also inhibited VEGF-induced proliferation, migration, and tube formation in human umbilical vein endothelial cells (HUVECs) and ex vivo angiogenesis in chick chorioallantoic membranes (CAMs). PROS treatment down-regulated the phosphorylation of VEGFR2, Src, and STAT3 in HUVECs. In the H460 xenograft model, subcutaneous injection of PROS down-regulated STAT3 and VEGF expression and caspase-3 activation. In Wang et al.’s study, either double-distilled water (ddH2O), 95% ethanol, or 1:1 water/ethanol extract of *S. miltiorrhiza* was added to two human oral squamous cell carcinoma (OSCC) cell lines, HSC-3 and OC-2, and it turned out that 95% ethanol extract of *S. miltiorrhiza* showed the greatest antioxidant and radical scavenging capabilities [36]. After treatment of HSC-3 cells with *S. miltiorrhiza* alcohol extract at doses of 10, 25, or 50 µg/mL for 48 or 72 h, significant decreases in the expression of X-linked inhibitor of apoptosis protein (XIAP) and survivin were observed but there were no changes in the levels of mitochondrial membrane potential (ΔΨm), antiapoptotic proteins (Bcl-2 and Bcl-xL), and proapoptotic proteins (Bax and Bcl-2 associated agonist of cell death [Bad]). In vivo, BALB/c NU mice xenografted with HSC-3 tumor were intraperitoneally injected with 50 or 100 mg/kg of *S. miltiorrhiza* extract for 34 days, which resulted in the suppression of tumor growth without any significant impacts on mouse body weights; as for biological markers, treatment with *S. miltiorrhiza* extract led to decreased expression of XIAP and survivin but not Bcl-2 family members. Yang et al. examined the antiproliferative effect of *S. miltiorrhiza* extract on oral cancer cells [37]. In vitro, 95% ethanol extract of *S. miltiorrhiza* was added to three OSCC cell lines SAS, SCC25, and Oec-ml (at doses between 0–30 µg/mL for 24 h) and six KB drug-resistant OSCC cell lines (at doses between 0–80 µg/mL for 24 h). In vivo, oral cancer SAS xenograft mice were administered with *S. miltiorrhiza* extract at a dose of 10 mg/kg for 32 days. Treatment with *S. miltiorrhiza* extract, both in vitro and in vivo, induced apoptosis, as evidenced by the increased active caspase-3 expression and decreased XIAP expression. According to Lee et al., acetonitrile extract of *S. miltiorrhiza* prevented the progression of prostate cancer cells through the generation of intracellular ROS generation [38]. Treatment with *S. miltiorrhiza* extract at doses of 5, 20, or 100 µg/mL for 24 or 48 h dose-dependently inhibited the growth of three prostate cancer cell lines (PC-3, LNCap, and DU-145) as measured by trypan blue assay. In addition, treatment with *S. miltiorrhiza* extract at a dose of 20 µg/mL for 24, 48, or 72 h induced cell cycle arrest at G1/S phase in PC-3 cells; it increased the protein expression of the cyclin-dependent kinase inhibitor p21 and decreased the protein expression of cyclin-dependent kinase 2 (CDK2), CDK4, and cyclin D1 protein. It also induced apoptosis in PC-3 cells as determined by TUNEL assay; it decreased the expression of anti-apoptotic Bcl-2 protein and increased the protein expression of apoptotic inducers such as caspase-9, caspase-3, and PARP. In vitro, PC-3 xenograft mouse model was injected with *S. miltiorrhiza* extract at doses of 100 mg/kg for 6 weeks. Both in vitro and in vivo, intracellular ROS generation was increased, which was considered to mediate the cytotoxic effect on prostate cancer cells. Sung et al. reported that 100% ethanol or 100% acetone extract of *S. miltiorrhiza* showed cytotoxicity against human cancer cell lines [39]. After treatment of AGS, A549, HCT116, LNCaP, and MCF-7 cells with *S. miltiorrhiza* extract (at doses of 5, 10, 20, 40 µg/mL for 24 h), p-JNK, p-ERK1/2, p-p38, cleaved-caspase-3, -7, -9, and cleaved poly ADP-ribose polymerase (c-PARP) expression were elevated. In contrast, it decreased the expression of nuclear p65, thereby inhibiting the progression of cancer cells. Wu et al. demonstrated that dichloromethane-methanol (1:1) extract of *S. miltiorrhiza* induced intrinsic apoptosis in various drug-sensitive and multidrug-resistant cancer cells [40]. Treatment with *S. miltiorrhiza* extract at doses of 3, 10, or 30 µg/mL for 1 h induced ROS production in CCRF-CEM cells. They also reported that *S. miltiorrhiza* extract induced apoptosis through caspases and a PARP-dependent pathway, as evidenced by the increased levels of cleaved caspase-3, -7, -9 and PARP upon treatment with *S. miltiorrhiza* extract at doses between 5–40 µg/mL. After treatment of CCRF-CEM cells with *S. miltiorrhiza* extract at a dose of 20 µg/mL for 24 h, TNF-α-induced translocation of p65 from cytoplasm to the nucleus was inhibited; when treated for 2 h, p-JNK, p-ERK1/2, and p-p38 expression were up-regulated. Therapeutic targets of *S. miltiorrhiza* in cancers are elucidated in Figure 1.

## 4. Cardiovascular Diseases

Cardiovascular disease (CVD) is the leading cause of mortality worldwide [41]. In 2017, CVD claimed 17.8 million lives globally, which was an increase of 21.1% from 2007. The global burden of CVD can be attributed to risk factors such as dietary risks, high systolic blood pressure, high body mass index, high total cholesterol level, high fasting plasma glucose level, tobacco smoking, and low levels of physical activity [42]. CVD includes all diseases and conditions of the heart and the blood vessels, but we here focus on ischemic heart disease (International Classification of Diseases (ICD): I20–25) and other forms of heart disease (ICD: I30-I52). The ischemic heart disease, also known as coronary heart disease, accounted for 49% of total global CVD burden in 2016 [43]. The clinical condition of ischemic heart disease includes angina, myocardial infarction, sudden death, and consequent chronic heart failure [44]. Among them, myocardial infarction (MI) is defined clinically as the presence of acute myocardial injury detected by abnormal cardiac biomarkers (i.e., cardiac troponin value above the 99th percentile upper reference limit) in the setting of evidence of acute myocardial ischemia; pathologically as myocardial cell death due to prolonged ischemia [45]. Prolonged ischemic condition leads to tremendous sudden death of cardiomyocytes primarily through cell necrosis, with partial involvement of apoptosis [46]. Necrotic cells and the damaged extracellular matrix tissue release danger signals, initiating an intense inflammatory response. Suppression and resolution of the inflammation, which are partly mediated by anti-inflammatory and pro-fibrotic cytokines (such as IL-10 and TGF-β_1_), are critical for optimal wound healing; dysregulation of them, in combination with overactive myocardial fibrosis, contribute to the pathogenesis of adverse left ventricular (LV) remodeling and eventually heart failure (HF) after MI [47].

### Cardiovascular Diseases and S. miltiorrhiza

**Figure 2:**
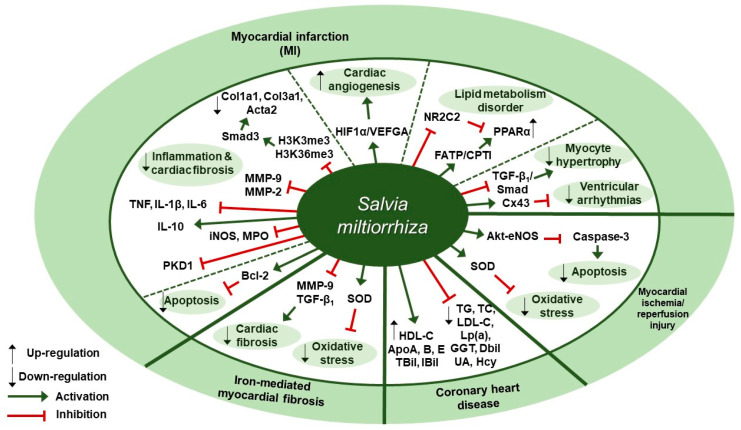
Therapeutic targets of *S. miltiorrhiza* in cardiovascular diseases. HIF1α, hypoxia-inducible factor 1α; VEGFA, vascular endothelial growth factor A; NR2C2, nuclear receptor subfamily 2 group C member 2; FATP, fatty acids transport protein; CPTI, carnitine palmitoyltransferase I; PPARα, peroxisome proliferator-activated receptor–α; Cx43, connexin 43; eNOS, endothelial nitric oxide synthase; SOD, superoxide dismutase; TG, triglycerides; TC, total cholesterol; LDL-C, low-density lipoprotein cholesterol; Lp(a), lipoprotein (a); GGT, gamma-glutamyl transpeptidase; DBil, direct bilirubin; UA, uric acid; Hcy, homocysteine; HDL-C, high-density lipoprotein cholesterol; ApoA, apolipoprotein A; TBil, total bilirubin; IBil, indirect bilirubin; MMP, matrix metalloproteinase; Bcl-2, B-cell lymphoma 2; PKD1, protein kinase D1 protein; iNOS, inducible nitric oxide synthase; MPO, myeloperoxidase; IL-10, interleukin-10; TNF, tumor necrosis factor; IL-1β, interleukin-1β; IL-6, interleukin-6; H3K4me3, H3K4 trimethylation; H3K36me3, H3K36 trimethylation; Col1a1, collagen type I alpha 1, Col3a1, collagen type III alpha 1; Acta2, actin alpha 2.

Several studies about protective effects of *S. miltiorrhiza* against cardiovascular diseases have been reported (Table 2). Liu et al. reported in a clinical trial that 3-month administration of Danshen pills, which were prepared from the extracts of *S. miltiorrhiza* and provided as 27 mg/pill, could lessen the chance of the coronary heart disease (CHD) risk by enhancing the biochemical indices of CHD patients [48]. Changes in the biochemical indices associated with CHD risk were assessed, such as lipid profile values (total cholesterol [TC], triglycerides [TG], low-density lipoprotein cholesterol [LDL-C], and high-density lipoprotein cholesterol [HDL-C]), apolipoproteins (ApoA, ApoB, and ApoE), lipoprotein (a)(Lp(a)), and markers of liver (γ-glutamyl transpeptidase [GGT], direct bilirubin [DBil], and indirect bilirubin [IBil]) and renal function (uric acid [UA] and homocysteine [Hcy]). The patients in the Danshen group received 10 *S. miltiorrhiza* pills three times a day for 3 months after three-month run-in period, whereas the control patients received placebo pills. At three-month follow-up, the Danshen group showed decreased levels of TC, TG, LDL-C, Lp(a), GGT, DBil, UA, and Hcy; on the contrary, they displayed increased levels of HDL-C, ApoA, ApoB, ApoE, TBil, and IBil. Zhang et al. examined the protective effect of *S. miltiorrhiza* injection against cardiac fibrosis induced by chronic iron overload (CIO) in mice [49]. Intraperitoneal injection of *S. miltiorrhiza* at doses of 3 or 6 g/kg decreased the heart weight-body weight coefficients, iron deposition levels, fibrotic area percentage, and hydroxyproline (Hyp) content. Further, *S. miltiorrhiza* injection reversed the changes in oxidative stress markers such as superoxide dismutase (SOD) activity and malondialdehyde (MDA) concentration. *S. miltiorrhiza* injection also dose-dependently lowered the protein expression levels of COL I, COL III, TGF-β_1_, and MMP-9. According to Ai et al., intraperitoneal *S. miltiorrhiza* injection at doses of 3 or 6 g/kg per day for 4 weeks had beneficial effects on impaired cardiac angiogenesis in a rat model of myocardial infarction via up-regulation of expression of key proangiogenic factors, hypoxia-inducible factor 1α (HIF1α) and vascular endothelial growth factor A (VEGFA) [50]. *S. miltiorrhiza* injection also improved ejection fraction (EF) and fractional shortening (FS), indicating its cardiac function-improving effect. Wang et al. showed that intramuscular administration of Danshen injection (DSI) at a dose of 1.5 mL/kg/d for 14 days could prevent left ventricular remodeling in a mouse model of heart failure induced by left anterior descending coronary artery (LAD) ligation [51]. DSI administration improved cardiac function as assessed by echocardiography parameters such as left ventricular ejection fraction (EF) and fractional shortening (FS); improved hemodynamic parameters (the maximum rate of LV pressure rise (dP/dt max) and fall (-dP/dt max), left ventricular end-diastolic pressure [LVEDP], volumes-related stroke volume, and cardiac output) and restored regular ventricular mass in a rat model of left anterior descending (LAD) coronary artery ligation. Further, DSI administration down-regulated MMP-2 protein expression, MMP-9, myeloperoxidase (MPO) and inducible nitric oxide synthase (iNOS) mRNA expression, indicating its anti-inflammatory effect; in contrast, DSI increased Bcl-2/Bax ratio, implying its anti-apoptotic effect. Yang et al. found that intraperitoneal administration of *S. miltiorrhiza* and *Carthamus tinctorius* extract (SCE) at a dose of 3 µL/g/day for 3 weeks attenuated cardiac dysfunction and myocardial fibrosis in a mouse model of LAD ligation [52]. Furthermore, SCE administration suppressed myocardial infarction (MI)-induced inflammation, as shown by the decreased levels of proinflammatory cytokines such as interleukin-1β (IL-1β), tumor necrosis factor (TNF), and interleukin-6 (IL-6), and the increased levels of anti-inflammatory cytokine interleukin-10 (IL-10). In vivo administration of SCE also had an inhibitory effect on the mRNA expression levels of fibrosis-related genes, such as collagen type I alpha 1 (Col1a1), collagen type III alpha 1 (Col3a1), actin alpha 2 (Acta2), and the expression levels of the proteins encoded by these genes, including type I collagen (COL I), COL III, and α-smooth muscle actin (SMA), respectively, which indicated SCE-induced attenuation of myocardial fibrosis in mice after MI. Further, they screened the proteins implicated in the TGF-dependent pathway and it turned out Smad3 in the SMAD family was suppressed by SCE administration. In vitro, SCE treatment reduced the TGF-β-induced increase in H3K4 trimethylation (H3K4me3) and H3K36 trimethylation (H3k36me3) at the Smad3 promoter region of cardiac fibroblasts, which led to decreased transcription of Smad3. Mao et al. demonstrated the extract of *S. miltiorrhiza* and its mixture with *Astragalus mongholicus* could inhibit myocardial fibrosis and ventricular remodeling by the regulation of protein kinase D1 (PKD1) protein in a rat model of myocardial infarction [53]. Oral administration of *S. miltiorrhiza* extract, alone or in combination with *Astragalus mongholicus* extract, at a dose of 20 mg/kg once a day for 8 weeks resulted in the improvement in systolic and diastolic functions as shown by the increased levels of left ventricular systolic pressure (LVSP), LVEDP, and ±dP/dt max; the maintenance of normal morphology and arrangement of cardiomyocytes as assessed by hematoxylin-eosin (H&E) staining; the reduction in contents of collagen fibers in myocardial tissues as evaluated by Masson’s trichrome staining; and the decreased expression level of protein kinase D1 (PKD1) as compared with the control group. Interestingly, combination of extracts of *S. miltiorrhiza* and *Astragalus mongholicus* led to better results as compared to the single use of either *S. miltiorrhiza* or *Astragalus mongholicus* extract. Wang et al. investigated the regulatory effect of the Danqi pill (DQP), which was prepared from water extracts of *S. miltiorrhiza* and *Panax notoginseng,* on MI-induced lipid metabolism disorder through activating fatty acids transport protein (FATP)-carnitine palmitoyltransferase I (CPTI) pathway and attenuating the inhibitory effect of nuclear receptor subfamily 2 group C member 2 (NR2C2) on peroxisome proliferator-activated receptor α (PPARα)-retinoid X receptor (RXR) pathway [54]. Oral administration of DQP at a dose of 1.5 mg/kg for 28 days in a SD rats model of myocardial ischemia improved cardiac function-related parameters, such as EF, FS, and left ventricular end-systolic diameter (LVEDs); decreased the levels of TG, LDL, ApoB, and hydroxy-3-methyl glutaryl coenzyme A reductase (HMGCR); and increased the levels of ApoA-I, cardiac FATP, and CPTI. Besides, DQP administration up-regulated PPARα-RXR pathway, as shown by the increased protein expression of PPARα and RXRs as well as the decreased protein expression of NR2C2. Ma et al. demonstrated that Danqi soft capsule (DQ), which was prepared from powdered water extracts of *S. miltiorrhiza* and *Panax notoginseng*, could inhibit infarct border zone (IBZ) remodeling and reduce susceptibility to ventricular tachyarrhythmias (VT) possibly via TGF-β_1_/Smad3 pathway in a rat model of post-myocardial infarction [55]. Oral administration of DQ at doses of 0.6, 0.9, or 1.2 g/kg for 4 weeks inhibited cardiac fibrosis in the IBZ as shown by the reduction in COL I and COL III expression levels; reversed Cx43 expression and distribution in the IBZ as assessed by Western blot and immunohistochemical staining; and prevented MI-induced myocyte hypertrophy in the IBZ as evidenced by the decreased mRNA expression levels of the hypertrophic marker atrial natriuretic peptide (ANP). Besides, DQ administration suppressed fibroblasts differentiation into myofibroblasts; it resulted in the decreased protein levels of α-SMA, TGF-β_1_, and p-Smad3. It also decreased the serum levels of brain natriuretic peptide (BNP), monocyte chemoattractant protein (MCP)-1 and TGF-β_1_. Xu et al. reported that 75% ethanol extraction of *S. miltiorrhiza* (IC_50_ = 86.5 µg/mL) could exert its cardiovascular protective and anti-inflammatory effects through inhibiting the activity of soluble epoxide hydrolase (sEH), a proinflammatory enzyme that converts its physiological substrate epoxyeicosatrienoic acids (EETs) to the less active dihydroxyeicosatrienoic acids (DHETs) [56]. They also tested which components of *S. miltiorrhiza* contributed to sEH inhibition based on a LC-MS/MS assay and discovered that tanshinone IIA and cryptotanshinone were potent (the inhibition constant [K_i_] = 0.87 µM) and medium (K_i_ = 6.7 µM) mixed-type inhibitors of sEH, respectively, and Salvianolic acid C was a moderate (K_i_ = 8.6 µM) noncompetitive inhibitor of sEH. Huang et al. showed *S. miltiorrhiza* and ligustrazine injection (SLI), prepared in a proportion of 1.2:60, had a beneficial effect on myocardial ischemia/reperfusion (I/R) and hypoxia/reoxygenation (H/R) injuries in mice subjected to coronary artery occlusion and 2 h reperfusion via Akt serine/threonine kinase (Akt)-endothelial nitric oxide synthase (eNOS) signaling pathway [57]. In vivo pretreatment with SLI at doses of 6.8, 20.4, or 61.2 mg/kg daily for 3 days improved cardiac function as illustrated by the increased LVSP and +dP/dt max, reduced the size of myocardial infarct, and mitigated the changes in myocardial histopathology. Besides, it decreased the blood serum levels of creatine kinase (CK), lactate dehydrogenase (LDH), and malondialdehyde (MDA) and increased the superoxide dismutase (SOD) levels. After in vitro treatment of H9C2 cells with SLI, apoptosis was inhibited, as evidenced by the decreased caspase-3 expression level and the increased Bcl-2/Bax ratio. Further, SLI pretreatment increased the phosphorylation of the survival kinase Akt at Ser473 and its downstream target eNOS following H/R. Therapeutic targets of *S. miltiorrhiza* in cardiovascular diseases are demonstrated in Figure 2.

## 5. Liver Diseases

When it comes to liver diseases, heavy alcohol consumption plays an important role in the occurrence of pathological conditions of the liver, such as steatohepatitis and liver fibrosis [58], while such conditions can also occur very commonly in non-alcoholic patients [59]. Alcoholic liver disease (ALD) and non-alcoholic fatty liver disease (NAFLD) are currently the most common etiologies of chronic liver disease (CLD) in the Western world, and their prevalence both are increasing worldwide [60]. Besides, the increasing alcohol misuse and prevalence of obesity and metabolic syndrome are raising the global burden of CLD [61]. ALD can be divided into 4 histological stages: steatosis, steatohepatitis, fibrosis not amounting to cirrhosis, and cirrhosis; progression to cirrhosis is most common in steatohepatitis (10% per year), followed by fibrosis (8%) and steatosis (3%). Mortality in hospitalized patients with steatohepatitis turned out to be even higher than that with cirrhosis, implying the need to treat the disease as high risk in an inpatient setting [62]. On the other hand, NAFLD encompasses two distinct conditions, one of which is non-alcoholic fatty liver (NAFL) and the other is non-alcoholic steatohepatitis (NASH); the latter includes diseases of varying severity, including fibrosis, cirrhosis, and hepatocellular carcinoma (HCC) [63]. Although there has been extensive research on understanding the pathophysiology of the diseases, a proven effective treatment for either ALD or NAFLD is yet to be found. Basically, the treatment strategy for ALD includes alcohol abstinence, nutritional support, steroids, anti-TNF therapy, antioxidants, and liver transplantation; for NAFLD, lifestyle changes (e.g., weight loss, dietary changes, and exercise), insulin sensitizers, lipid lowering agents, antioxidants, and anti-inflammatory agents [64].

### Liver Diseases and S. miltiorrhiza

**Figure 3:**
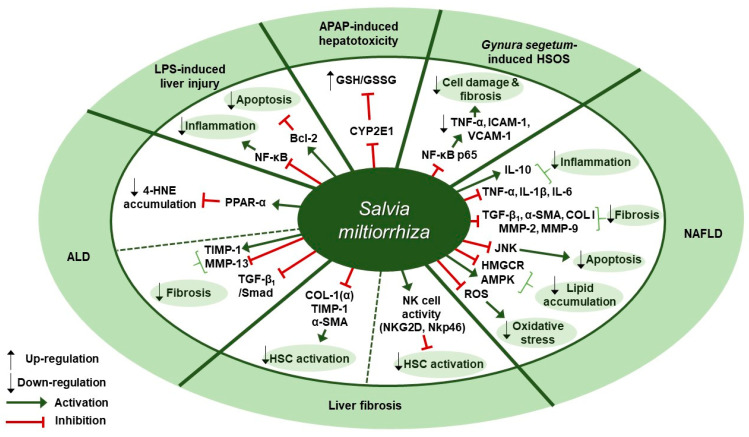
Therapeutic targets of *S. miltiorrhiza* in liver diseases. ALD, alcoholic liver disease; LPS, lipopolysaccharide; APAP, acetaminophen; HSOS, hepatic sinusoidal obstruction syndrome; NAFLD, non-alcoholic fatty liver disease; CYP2E1, cytochrome P450 2E; GSH/GSSG ratio, glutathione/glutathione disulfide ratio; NF-κB, nuclear factor kappa-light-chain-enhancer of activated B cells; TNF-α, tumor necrosis factor-α; ICAM-1, intercellular adhesion molecule-1; VCAM-1, vascular cell adhesion molecule 1; IL-10, interleukin 10; IL-1β, interleukin 1 beta; IL-6, interleukin -6; TGF-β1, transforming growth factor beta 1; α-SMA, alpha-smooth muscle actin; COL I, type I collagen; MMP, matrix metalloproteinase; JNK, c-Jun N-terminal kinase; HMGCR, 3-hydroxy-3-methyl-glutaryl-coenzyme A reductase; AMPK, AMP-activated protein kinase; ROS, reactive oxygen species; NKG2D, natural killer group 2D; NKp46, natural killer p46; HSC, hepatic stellate cell; TIMP-1, tissue inhibitor of metalloproteinase-1; PPAR-α, peroxisome proliferator-activated receptor alpha; 4-HNE, 4-hydroxynonenal; Bcl-2, B-cell lymphoma 2.

Several studies about therapeutic effects of *S. miltiorrhiza* in liver diseases have been reported (Table 3). Ding et al. presented experimental evidence for the protective effect of Danshen injection (DSI) on alcoholic liver disease (ALD) through the activation of peroxisome proliferator-activated receptor alpha (PPARα) and subsequent induction of 4-hydroxynonenal (4-HNE) degradation [65]. Intraperitoneal administration of DSI at a dose of 3 g/kg daily for 4 weeks in a mouse model of ALD induced by 5-week ethanol-containing diet attenuated pathological changes of liver as assessed by plasma ALT levels, hepatic triglyceride contents, and liver weight. DSI increased the protein and mRNA expression of PPARα and the mRNA expression of its downstream target genes carnitine palmitoyltransferase 1 (CPT-1) and CPT-2 in liver tissues of mice. They further discovered that the hepatoprotective effect of DSI was associated with 4-HNE degradation which was mediated by PPAR-α activation by DSI. In vitro pretreatment with DSI at doses of 100 or 200 µg/mL for 2 h inhibited 4-HNE-induced cell death in HepG2 cells, as evidenced by the reduced LDH release. In addition, treatment of NCTC1469 cells with 150 µg/mL of DSI for 2 h resulted in reduced intracellular 4-HNE accumulation after exogenous 4-HNE exposure, whereas pretreatment with GW6471, a PPARα antagonist, abolished the inhibitory effect of DSI on 4-HNE accumulation. Li et al. showed that a herbal mixture extract PSSS composed of *Pueraria lobata* (40%), *S. miltiorrhiza* (25%), *Schisandra chinensis* (20%), and *Silybum marianum* (15%) had beneficial effect on alcoholic liver fibrosis (ALF) in rats possibly via the TGF-β_1_/Smad signaling pathway [66]. Sprague Dawley (SD) rats were treated with PSSS at doses of 0.333, 0.667, or 1 g/kg for 30 days after fed with Lieber-Decarli alcohol liquid diet and CCl4 injection. PSSS treatment improved histopathological lesions in the liver and decreased the serum levels of liver fibrosis indicators (hyaluronan [HA], laminin [LM], and hydroxyproline [67]). Further, the decreased expression of hepatic MMP-13 and the increased expression of hepatic tissue inhibitor of metalloproteinase (TIMP)-1 was reversed by PSSS administration in mice. It also down-regulated TGF-β_1_ protein expression at doses of 0.667 or 1 g/kg and protein and mRNA expression of p-Smad2 and p-Smad3 at all three doses, whereas it up-regulated Smad7 protein expression at all three doses. Zhou et al. found that treatment with *S. miltiorrhiza* extract could inhibit acetaminophen (APAP)-induced hepatotoxicity through its antioxidant activity and inhibition of cytochrome P450 family 2 subfamily E member 1 (CYP2E1) [68]. One hour of *S. miltiorrhiza* extract pretreatment enhanced cell viability in APAP-stimulated primary SD rat hepatocytes at doses of 0.25 and 1 mg/mL, as shown by 5.6-fold increase in the half maximal effective concentration (EC_50_ > 40 mM) which was detected in MTT assay. In the alamar blue assay, *S. miltiorrhiza* extract (EC_50_ = 40.61±6.82 mM) increased the mitochondrial metabolic activity by 5.3-fold. Further, *S. miltiorrhiza* extract (IC_50_ = 1.07 mg/mL) concentration dependently inhibited CYP2E1-mediated formation of 6-hydroxy-chlorzoxazone in SD rat liver microsomes and turned out to be a mixed type inhibitor of CYP2E1. Besides, in vitro treatment with *S. miltiorrhiza* extract at a dose of 1 mg/mL exerted an antioxidant effect in rat hepatocytes, as can be seen by the fact that it prevented the depletion of total glutathione (total GSH) induced by 24-h APAP treatment and that it increased the level of glutathione/glutathione disulfide ratio (GSH/GSSG ratio) even higher than 1 mM N-acetyl-cysteine (NAC), the positive control. Yang et al. showed that *S. miltiorrhiza* at doses of 100 or 200 mg/kg improved liver function and attenuated endothelial cell damage in Kunming mice model of *Gynura segetum-*induced hepatic sinusoidal obstruction syndrome (HSOS) [69]. Further, *S. miltiorrhiza* decreased the expression of TNF-α, vascular cell adhesion molecule 1 (VCAM-1), intercellular adhesion molecule-1 (ICAM-1), and NF-κB p65, thereby inhibiting HSOS in a dose-dependent manner. According to Gao et al., Danhong injection (DHI), a mixture extract of *S. miltiorrhiza* and *Carthamus tinctorius* (5:2), at a dose of 3 g/kg (crude drugs weight/body weight) for 30 min showed anti-inflammatory, anti-oxidative, and anti-apoptotic effects in an lipopolysaccharide (LPS, 16 mg/kg)-induced mouse model of acute hepatic injury [70]. The intraperitoneal administration of DHI ameliorated the aberrant changes in biochemical markers, including alanine transaminase (ALT), aspartate transaminase (AST), and total bilirubin (TBil) levels in serum and malondialdehyde (MDA) and glutathione-S-transferase (GST) levels in liver homogenates. Further, DHI inhibited the expression of TNF-α, IL-6, caspase-3, and Bax and the phosphorylation of the inhibitor of nuclear factor kappa B α (IκBα) and nuclear factor-κB (NF-κB) p65, whereas it significantly increased Bcl-2 expression. Parajuli et al. examined the effect of PF2401-SF, a standardized and purified ethanol extract of *S. miltiorrhiza*, on gene and protein expression of liver fibrosis-related factors in mice with liver fibrosis induced by thioacetamide (TAA) [71]. Oral administration of PF2401-SF at doses of 1 or 2.5 mg/kg for 12 weeks resulted in improvement in serum levels of AST and ALT, reduction in fibrous tissue deposition as observed with H&E staining, and down-regulation of COL I (α), TIMP-1, and α-SMA expression. Given that α-SMA is a vital marker for hepatic stellate cells (HSCs) activation, the anti-fibrotic effect of PF2401-SF was suggested to be attributed to the decreased HSC activation. PF2401-SF had hepatoprotective and anti-fibrotic effects on liver fibrosis. According to Peng et al., 90% ethanol extract of *S. miltiorrhiza* was reported to have an anti-fibrotic effect on carbon tetrachloride (CCl_4_)-induced liver fibrosis in mice possibly via the activation of hepatic natural killer cells [72]. Administration of *S. miltiorrhiza* extract at doses of 1.5 and 3.0 g/kg for 4 weeks ameliorated CCl_4_-induced liver injury and fibrosis in mice, as assessed by H&E and Sirius Red staining, serum ALT and AST levels, and Hyp contents. Besides, in vivo *S. miltiorrhiza* extract administration at a dose of 3 g/kg up-regulated the frequency of NK cells and the expression of their active receptors, natural killer group 2D (NKG2D) and natural killer p46 (Nkp46). In vitro pre-incubation of NK cells with *S. miltiorrhiza* extract at a dose of 50 µg/mL for 16 h in the presence of interleukin-2 (IL-2) resulted in the increased expression of NKG2D and interferon gamma (IFN-γ), as well as the increased activation of primary NK cells. *S. miltiorrhiza* extract pretreatment for 16 h enhanced the inhibitory function of NK cells on the activation of hematopoietic stem cells (HSCs) (in this case, JS-1 cells) as assessed by the decreased level of α-SMA and the increased level of RAE-1ε, the NK cell stimulatory ligand on the surface of JS-1 cells. Lee et al. showed that a standardized ethanol extract of *S. miltiorrhiza* could inhibit the development and progression of non-alcoholic steatohepatitis (NASH) [73]. In vivo, *S. miltiorrhiza* extract administration at doses of 0.5 or 1 mg/kg every other day for 4 or 6 weeks improved histopathological changes such as the inflammation and fibrosis of hepatocytes in a mouse model of NASH induced by a methionine-choline deficient (MCD) diet, as evaluated by H&E and Sirius Red staining. In vivo *S. miltiorrhiza* extract administration for 4 weeks also down-regulated hepatic protein expression of TNF-α and COL I and mRNA expression of NASH-related specific genes such as TNF-α, TGF-β_1_, IL-1β, C-reactive protein (CRP), α-SMA, COL I, MMP-2, MMP-9. In vitro treatment with *S. miltiorrhiza* extract produced similar results in TGF-β_1_-induced LX-2 human hepatic stellate cells. Besides, pretreatment with *S. miltiorrhiza* extract at doses of 0.1, 1, 10, or 100 µg/mL for 30 min ameliorated intracellular ROS production in LX-2 cells later treated with hydrogen peroxide (H_2_O_2_) for 15 min, suggesting its antioxidant role. Lee et al. demonstrated the administration of CG^plus^, a standardized water extract of *Artemisia iwayomogi*, *Amomum xanthioides*, and *S. miltiorrhiza*, exerted a protective effect in a C57/BL6J mice model of NASH [74]. CG^plus^ administration at doses of 50, 100, and 200 mg/kg for 5 days down-regulated the protein and mRNA expression of pro-inflammatory cytokines (TNF-α, IL-1β, IL-6) and up-regulated the expression of anti-inflammatory cytokine IL-10. In addition, it increased the mRNA expression of lipolytic molecules (AMP-activated protein kinase [AMPK] and acyl-CoA dehydrogenase long chain [ACADL]) and the protein expression of p-AMPK, whereas it decreased the mRNA expression of lipogenic molecules including 3-hydroxy-3-methyl-glutaryl-coenzyme A reductase [HMGCR] and the protein expression of phosphorylated sterol regulatory element-binding protein 1 (SREBP-1). According to Feng et al., Jianpi Huoxue formula (JPHX) composed of *Atractylodes macrocephal*, *S. miltiorrhiza*, *Radix Paeonia alba*, *Rhizoma alismatis*, and *Fructus Schisandrae chinensis* had beneficial effects on nonalcoholic fatty liver disease (NAFLD) by regulating lipid accumulation, inflammation, fibrosis, and apoptosis [75]. In vivo administration of JPHX at doses of 0.6, 1.21, or 2.42 g/kg once a day for 8 weeks ameliorated hepatic injury and inflammation, as shown by the decreased AST and ALT levels and TNF-α expression, and inhibited the accumulation of hepatic triglycerides (TG) and total cholesterol (TC) in MCD diet-fed rats. Further, it suppressed hepatic fibrosis, as indicated by the decreased expression levels of profibrotic genes such as MMP-9 and COL I. It was noteworthy that JPHX down-regulated hepatic apoptosis, as assessed by terminal deoxynucleotidyl transferase-mediated dUTP nick end labeling (TUNEL) assay, and a significant decrease in the expression of p-JNK was observed. Therapeutic targets of *S. miltiorrhiza* in liver diseases are presented in Figure 3.

## 6. Nervous System Diseases

As for nervous system diseases, neurodegenerative diseases such as Alzheimer’s disease and cerebrovascular diseases such as ischemic stroke are two major categories of neurologic disorders and there has been a growing notion that the two are closely intertwined, with overlap in pathology and risk factors [76,77,78,79]. Alzheimer’s disease is one of the most challenging diseases to cure and is the major cause of dementia [80]. Its pathophysiology has been associated not only with amyloid-β (Aβ) plaques, hyper-phosphorylated tau neurofibrillary tangles, and neuronal loss, all of which are classical pathological hallmarks of the disease [81]; but also with the cerebral blood flow shortfalls and blood–brain-barrier dysfunction, which are being given considerable attention as contributing factors to its pathogenesis [82]. There are currently five FDA-approved drugs for the treatment of Alzheimer’s disease: rivastigmine, galantamine, donepezil, memantine, and memantine combined with donepezil [83]. Memantine, a non-competitive NMDA antagonist, proved to improve learning and memory by restoring homeostasis in the glutamatergic system [84]. The rest of them besides memantine are cholinesterase inhibitors (ChEIs) and are first-line, symptomatic treatments of the disease, with gastrointestinal side effects as the most common adverse reactions [85]. Ischemic stroke is by far the most common type of stroke, which ranks fifth among all causes of mortality. The stroke of this type is also referred to as brain ischemia or cerebral ischemia, and it accounts for 87% of all strokes [42]. When there is a reduction in cerebral blood flow due to a thrombus formation in the brain vasculature, ischemic stroke occurs [86]. Ischemic stroke is the major factor for vascular dementia, which accounts for around 10% of all dementia cases [87]. Post-stroke cognitive impairment is prevalent worldwide and the underlying mechanisms include vascular cognitive impairment owing to neuroanatomical lesions in such areas as hippocampus and white matter [88]. There are several other factors under investigation that make up the pathophysiology of ischemic stroke, such as post-stroke inflammation [89], apoptosis [90], and more specifically, protein kinase C activation [91].

### Nervous System Diseases and S. miltiorrhiza

**Figure 4:**
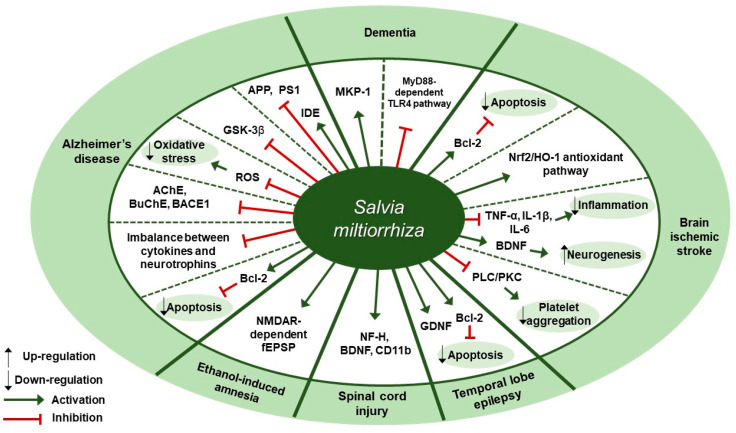
Therapeutic targets of *S. miltiorrhiza* in nervous system diseases. MKP-1, mitogen-activated protein kinase-1; MyD88, myeloid differentiation primary response 88; TLR4, toll-like receptor 4; Bcl-2, B-cell lymphoma 2; Nrf2, nuclear factor erythroid 2-related factor 2; HO-1, heme oxygenase-1; TNF-α, tumor necrosis factor-α; IL-1β, interleukin-1β; IL-6, interleukin-6; BDNF, brain-derived neurotrophic factor; PLC, phospholipase; PKC, protein kinase C; GDNF, glial cell-derived neurotrophic factor; NF-H, neurofilament 200; CD11b, cluster of differentiation molecule 11B; NMDAR, N-methyl-d-aspartate receptor; fEPSP, field excitatory postsynaptic potential; AchE, acetylcholinesterase; BuChE, butyrylcholinesterase; BACE1, β-secretase; ROS, reactive oxygen species; GSK-3β, glycogen synthase kinase-3β; APP, amyloid precursor protein; PS1, presenilin-1; IDE, insulin-degrading enzyme.

Several studies about neuroprotective effects of *S. miltiorrhiza* have been reported (Table 4). According to Yu et al., pre-treatment of SH-SY5Y cells with *S. miltiorrhiza* extract at doses of 0.01, 0.1, or 0.2 mg/mL for 2 h attenuated Aβ_25-35_-induced cell death through the inhibition of oxidative stress and mitochondria-dependent apoptotic pathway [92]. *S. miltiorrhiza* extract decreased the intracellular reactive oxygen species (ROS) levels, cleaved caspase-3 and cytosolic cytochrome c expression, and Bax/Bcl-2 ratio. Paudel et al. reported that *S. miltiorrhiza* extract significantly inhibited the expression of glycogen synthase kinase-3β (GSK-3β) with IC_50_ values of 7.77±1.38 [93]. According to Ozarowski et al., subchronic (28-fold) oral administration of 200 mg/kg *S. miltiorrhiza* extract for 28 days led to an improvement in long-term memory of rats as assessed by passive avoidance test, partially through inhibition of acetylcholinesterase (AChE) activity [94]. *S. miltiorrhiza* extract administration decreased AChE activity by 47% in the frontal cortex and by 55% in the hippocampus and decreased its relative mRNA expression in rat brain cortex homogenates by 41% (*p* < 0.01) compared to the control group. Besides, it down-regulated the mRNA expression of butyrylcholinesterase (BuChE) by 48% (*p* < 0.05) and β-secretase (BACE1) by 48% in the cortex, but there was no treatment-induced significant difference in the hippocampus of rat brain. Teng et al. reported that compound Danshen tablet (CDT) composed of *S. miltiorrhiza*, *Panax notoginseng*, and *Borneol* (450:141:8) had beneficial effects on cognitive function in Aβ_25-35_-induced mice model when administered at doses of 0.405 or 0.81 g/kg for 7 days [95]. CDT elevated choline acetyltransferase (CHAT) and receptor of activated protein kinase C1 (RACK1) levels and restored the balance between cytokines (interleukin-6 [IL-6] and tumor necrosis factor-α [TNF-α]) and neurotrophins (brain-derived neurotrophic factor [BDNF]) in the hippocampus and cortex of brain. Similarly, Liu et al. demonstrated treatment with Danshen tablet of the same composition and proportion at a dose of 520 mg/kg for 14 days enhanced spatial learning and memory in Aβ_25-35_ peptide-induced rat model by increasing the expression levels of insulin-degrading enzyme (IDE) and decreasing the expression levels of amyloid precursor protein (APP) and presenilin-1 (PS1) [96]. Kim et al. demonstrated the administration of *S. miltiorrhiza* extract at a dose of 50 mg/kg for 2 weeks in combination with 1 × 10^6^ mesenchymal stem cells (MSC) resulted in synergetic therapeutic effects on the recovery of ischemic stroke in a rat middle cerebral artery occlusion model via enhancing the anti-apoptotic and survival ability of MSCs under hypoxic conditions [97]. Treatment with *S. miltiorrhiza* extract up-regulated the expression of Bcl-2, phospho-protein kinase B (p-Akt), and phospho-extracellular signal-regulated kinase (p-ERK) and down-regulated the expression of Bax and caspase-3. According to Wang et al., salvianolate lyophilized injection (SLI), which was obtained by 80% ethanol extraction of *S. miltiorrhiza*, protected against cerebral ischemic injury in type 1 diabetic rats subjected to intraluminal middle cerebral artery occlusion through down-regulation of inflammatory factors and up-regulation of nuclear factor erythroid 2-related factor 2 (Nrf2)/heme oxygenase-1 (HO-1) antioxidant pathway [98]. Administration of SLI at doses of 5.25, 10.5, or 21 mg/kg for 15 days attenuated neural cell injury in the ischemic penumbra of cortex as assessed by H&E staining. Further, it down-regulated receptor for advanced glycation endproducts (RAGE), matrix metalloproteinase-9 (MMP-9), cyclooxygenase-2 (COX-2), TNF-α, and intercellular adhesion molecule-1 (ICAM-1) expression and up-regulated HO-1, NAD (P) H quinine oxidoreductase (HQO-1), and Nrf2 expression. Fei et al. demonstrated that supercritical CO_2_ and 95% ethanol extract of *S. miltiorrhiza* could alleviate permanent cerebral ischemic injury induced by permanent middle cerebral artery occlusion (pMCAO) in rats via activating phospholipase (PLC)/protein kinase C (PKC) signaling pathway [99]. Specifically, in vivo experiments were conducted at doses of 3.75, 7.5, or 15 mg/kg once daily for 3 days to assess the effect of *S. miltiorrhiza* extract on thrombosis formation and platelet aggregation, while in vitro experiments were conducted on platelets from rat plasma at doses of 0.44, 4.4, or 44 mg/L for 10 min to assess platelet aggregation, thromboxane A_2_ (TXA_2_) release, and the activation of platelet activating proteins (PLCβ3, PKC). Treatment with *S. miltiorrhiza* extract at different doses inhibited thrombosis formation and platelet aggregation, inhibited TXA_2_ release, and down-regulated p-PLCβ3 and p-PKC expression. According to Zhang et al., the water extract of *S. miltiorrhiza*, *Ligusticum chuanxiong*, and *Carthamus tinctorius*, orally administered into mice with middle cerebral artery occlusion (MCAO), could help to prevent acute cerebral ischemic injury and recover cognitive impairment through inhibition of microenvironmental inflammation and activation of neurogenesis in the hippocampus [100]. Pretreatment with the mixture extract at a dose of 20 g/kg for 5 days had a preventive effect in MCAO mouse model by reversing the up-regulation of Bax, interleukin-1β (IL-1β), IL-6, and TNF-α expression and the down-regulation of Bcl-2 expression. Chronic treatment at a dose of 20 g/kg for 28 days attenuated ischemia-induced spatial memory impairments, as evidenced by the decreased escape latency test in the Morris water maze test, and it greatly increased neurogenesis and BDNF expression. Cai et al. showed that an intraperitoneal injection of *S. miltiorrhiza* extract at a dose of 5 mL/kg/day for 4 weeks could ameliorate the learning and memory decline of diabetic rats, as shown by the improved escape latency and platform-finding frequency in the Morris water maze test and the increased mitogen-activated protein kinase-1 (MKP-1) protein expression under the hyperglycemic condition [101]. According to Kim et al., the oral administration of *S. miltiorrhiza* extract could protect against the damage to the white matter and hippocampus in bilateral common carotid artery occlusion (BCCAo) rat model possibly via myeloid differentiation primary response 88 (MyD88)-dependent toll-like receptor 4 (TLR4) pathway [102]. *S. miltiorrhiza* extract administration at a dose of 200 mg/kg/day for 22 days attenuated the BCCAo-induced increase in TNF-α, IL-1β, IL-6, TLR4, and MyD88 and the decrease in myelin basic protein (MBP). Park et al. demonstrated that the ethanol extract of *S. miltiorrhiza* alleviated ethanol-induced behavioral and synaptic deficits in mice via regulating N-methyl-d-aspartate (NMDA) receptor-dependent synaptic transmission [103]. *S. miltiorrhiza* (200 mg/kg), orally administered 30 min prior to ethanol treatment, protected against the impairment of object recognition memory. Twenty minutes of *S. miltiorrhiza* perfusion at doses of 10 or 100 µg/mL before ethanol perfusion substantially mitigated ethanol-induced long-term potentiation (LTP) and NMDA receptor-mediated field excitatory post-synaptic potential (fEPSP) deficits in the hippocampal slices. According to Zhang et al., 75% ethanol extract of *S. miltiorrhiza* exerted a beneficial effect on the recovery of locomotor function after spinal cord injury (SCI) in rats [104]. *S. miltiorrhiza* extract administration at a dose of 12.5 g/kg for 8 days improved motor function of SCI rats, as assessed by the Basso, Beattie, and Bresnahan locomotor scale, and ameliorated histopathological changes in the injured spinal cord, as observed with H&E staining. Besides, it up-regulated the expression of neurofilament 200 (NF-H) (a mature neuron marker), BDNF, and cluster of differentiation molecule 11B (CD11b) (a microglia marker). These results were indicative of axonal regeneration, spinal cord repair, and microglial cells activation, respectively. Jia et al. reported that compound Danshen dripping pills (CDDP) composed of *S. miltiorrhiza*, *Panax ginseng*, and *Borneol*, alone or in combination with carbamazepine (CBZ), had protective and cognitive improving effects in a kainic acid-induced temporal lobe epilepsy (TLE) rat model [105]. The intragastric administration of CDDP at a dose of 85 mg/kg for 90 days alone or in combination with CBZ at a dose of 100 mg/kg attenuated cognitive impairment as assessed by the Morris water maze test. Further, it increased glial cell line-derived neurotrophic factor (GDNF) expression and the Bcl-2/Bax ratio in the hippocampal CA3 region, thereby minimizing the neuronal cell death. Therapeutic targets of *S. miltiorrhiza* in nervous system diseases are shown in Figure 4.

## 7. Discussion

*Salvia miltiorrhiza* Bunge (*S. miltiorrhiza*), also known as dansam in Korean and danshen in Chinese, is a medicinal herb which belongs to genus *Salvia* of Lamiaceae family [2]. In Korea, it is known to be effective in removing blood stasis and enriching the blood according to the original text of Donguibogam (a classic written in the 17th century) [106]. It has been widely used for the treatment of various diseases, but there are only a few review articles addressing its multi-functional therapeutic potentials. Yan et al. reviewed its traditional medicinal uses and pharmacological properties based on articles reporting on the beneficial effects of compounds isolated from *S. miltiorrhiza* [107]. We, however, aimed to gather data on the efficacy of the whole herb extract of *S. miltiorrhiza* to obtain insights into how *S. miltiorrhiza* extract as a whole exerted its therapeutic effects in various diseases.

### 7.1. S. miltiorrhiza Exhibits Anti-cancer Activity by Inducing Apoptosis in Cancer Cells

In terms of cancer therapeutics, one major strategy is to induce apoptosis in cancer cells by triggering the key components of cell death signaling pathway such as Bcl-2 family of proteins and the caspases [108], or modulating the MAPK families [109]. Indeed, in most of the studies mentioned earlier in our review, induction of apoptosis in cancer cells was the major goal of each study and they mostly explored the detailed mechanisms by which *S. miltiorrhiza* extract exerted its apoptotic effects. In doing so, different doses of *S. miltiorrhiza* extract alone or mixed with other herb extracts were tested as a therapeutic agent in cancer cell lines or mouse xenograft models. Among them, some studies offered conflicting results regarding the mechanism by which *S. miltiorrhiza* extract induced apoptosis. For example, 95% ethanol extract of *S. miltiorrhiza* at doses between 10–50 µg/mL for 48 or 72 h induced apoptosis in OSCC cell lines, by down-regulating the expression of XIAP and survivin, two members of the inhibitor of apoptosis protein (IAP) family; however, treatment with *S. miltiorrhiza* extract did not change the levels of antiapoptotic proteins Bcl-2 and Bcl-xL and the proapoptotic proteins Bax and Bad, as well as apoptosis-related mitochondrial membrane potential [36]. In contrast, treatment with methanol extract of *S. miltiorrhiza* at doses between 10–40 µg/mL for 24 h decreased the expression of Bcl-2 and Bcl-xl and increased the expression of Bax in Glc-82 cells; in addition, treatment with 20 and 40 µg/mL of *S. miltiorrhiza* extract increased the levels of cleaved caspase-9, caspase-3, and PARP1, suggesting the possibility of the involvement of the mitochondrial apoptotic pathway in *S. miltiorrhiza* extract-mediated cytotoxicity [34]. Wu et al.’s study supported this finding in that the treatment with *S. miltiorrhiza* extract induced apoptosis in CCRF-CEM cells through the intrinsic apoptotic signaling pathway, as evidenced by the increased expression of cleaved caspase-9, -7, -3, and PARP [40]. Lee et al. reported that the treatment with *S. miltiorrhiza* extract activated caspase-9, caspase-3, and PARP and did not impact the activity of caspase-8, but they concluded that the *S. miltiorrhiza* extract induced apoptosis exclusively through the extrinsic apoptotic signaling pathway [38], which seems to be a mistake since caspase-9 is considered as an upstream initiator required for the mitochondrial apoptotic pathway, or intrinsic apoptotic pathway, while caspase-8 as an initiator caspase that mediates the extrinsic apoptotic pathway [110]. On the other hand, *S. miltiorrhiza* extract has been shown to modulate the MAPK families, which consist of multiple kinases altered in cancers and thereby have been considered a promising target [109]. According to Kim et al., treatment with *S. miltiorrhiza* extract at a dose of 50 µg/mL for 24 h specifically inhibited the MAPK/AP-1 signaling pathway in TPA-induced breast cancer cells and did not influence the phosphorylation of inhibitory κ B kinase (IKK) α/β and IκBα subunit, degradation of IκBα, or nuclear translocation of NF-κB p65 [30]. However, in Wu et al.’s study, treatment with *S. miltiorrhiza* extract at a dose of 20 µg/mL for 24 h inhibited the nuclear translocation of NF-κB p65 in CCRF-CEM cells, while down-regulation of ERK and JNK was observed in both studies [40]. These conflicting results warrant further research into the precise mechanism implicated in the anti-apoptotic effect of *S. miltiorrhiza*.

### 7.2. S. miltiorrhiza Exerts Anti-inflammatory and Anti-fibrotic Effects in Modulating Cardiovascular Diseases

One major component of cardiovascular diseases (CVD) is the ischemic heart disease (IHD) [43], which encompasses a broad spectrum of clinical conditions including angina pectoris, unstable angina, and myocardial infarction (MI), all of which are characterized by myocardial ischemia [111]. A possible treatment approach to IHD is anti-inflammation in coronary artery [112], as illustrated by studies on IL-1β as a therapeutic target for the treatment of atherosclerotic vascular disease [113,114]. In this perspective, *S. miltiorrhiza* extract has recently been addressed in several studies as a potential anti-inflammatory agent in a mouse model of MI induced by LAD ligation; specifically, administration of *S. miltiorrhiza* extract reduced MI-induced inflammation, as evidenced by the decreased expression of proinflammatory cytokines such as IL-1β, TNF, and IL-6 and the increased expression of anti-inflammatory cytokines IL-10 in Yang et al.’s study [52] and the decreased expression of iNOS and MPO in Wang et al.’s study [51]. Cardiac fibrosis is a common histopathological phenomenon in IHD and is characterized by the increased accumulation of extracellular matrix (ECM) that impairs cardiac function [115]. It has been suggested targeting fibrosis would be a promising therapeutic option for the treatment of chronic heart failure or other CVDs [116]. Most of the studies which were included in our review tested whether *S. miltiorrhiza* extract could inhibit cardiac fibrosis and ventricular remodeling. Various measures to assess cardiac fibrosis were used, such as immunohistochemistry and Masson’s trichrome staining. For example, *S. miltiorrhiza* extract administration dose-dependently decreased the expression of COL I and COL III as assessed by immunohistochemistry and reduced areas of fibrotic heart tissue as observed with Masson’s stain [49]. Two studies further examined the effect of *S. miltiorrhiza* extract on the expression levels of the profibrotic cytokine TGF-β, which is a major mediator implicated in fibrotic remodeling of the heart and is thought to drive fibroblast-myofibroblast differentiation [117]; in both studies, down-regulation of TGF-β expression was observed, but different mechanisms were proposed regarding the anti-fibrotic effects of *S. miltiorrhiza* extract [49,55].

### 7.3. S. miltiorrhiza Exerts Several Effects in Modulating Liver Diseases

To date, there is no proven effective treatment option for alcoholic liver disease (ALD) and non-alcoholic fatty liver disease (NAFLD), both of which are chronic liver diseases with a shared pathological spectrum ranging from simple steatosis to hepatitis to cirrhosis to hepatocellular carcinoma [64]. Natural compounds from plants have been suggested as an alternative to treat liver pathology and their hepatoprotective effects have been extensively investigated in terms of pharmacological properties including antioxidant, anti-inflammatory, anti-fibrotic, anti-apoptotic, and hepatoprotective activities [118]. Since oxidative stress plays a vital role in the pathogenesis of many liver diseases including ALD and NAFLD [119], the application of natural antioxidants could be a rational strategy for the treatment of liver diseases [120]. According to recent studies mentioned earlier in our review, *S. miltiorrhiza* could be a natural resource of antioxidants, as illustrated by Zhou et al.’s study [68] where *S. miltiorrhiza* extract attenuated the loss of total glutathione (GSH) by inhibiting CYP2E1 and preserved redox status expressed as GSH/GSSG level in rat hepatocytes. Lee et al. also demonstrated the antioxidant role of *S. miltiorrhiza* extract as indicated by the decreased intracellular ROS generation in LX-2 cells after treatment with *S. miltiorrhiza* extract at doses of 0.1, 1, 10, or 100 µg/mL for 30 min [73]. On the other hand, several studies pointed out other therapeutic properties of *S. miltiorrhiza* extract, including anti-inflammatory and anti-fibrotic activities. For example, the anti-fibrotic effect of *S. miltiorrhiza* extract in a mouse model of thioacetamide-induced liver fibrosis was demonstrated in Parajuli et al.’s study, where *S. miltiorrhiza* extract administration at doses of 1 or 2.5 mg/kg for 12 weeks reduced fibrous tissue deposition and down-regulated the expression of central markers of fibrosis including COL I, TIMP-1, and α-SMA [71]. While reduced activation of hepatic stellate cells (HSC) was proposed as a possible mechanism for the anti-fibrotic activity of *S. miltiorrhiza* extract in Parajuli et al.’s study, Peng et al. suggested that the administration of *S. miltiorrhiza* extract at doses of 1.5 or 3 g/kg for 4 weeks attenuated CCL_4_-induced liver injury and fibrosis in mice through the activation of hepatic natural killer cells, which also contributed to HSC deactivation [72]. On the other hand, a few studies reported on the hepatoprotective effects of *S. miltiorrhiza* extract via modulating the activity of the NF-κB family of transcription factors, which have been implicated in liver injury with regard to its role in liver homeostasis and the regulation of inflammation, fibrosis, and carcinogenesis [121]. For instance, *S. miltiorrhiza* at doses of 100 or 200 mg/kg ameliorated endothelial cell damage in a rat model of *Gynura segetum*-induced hepatic sinusoidal obstruction syndrome (HSOS) when it down-regulated NF-κB p65, one of five components that form the NF-κB [69]. Inhibition of NF-κB by *S. miltiorrhiza* extract was associated with the decreased expression of proinflammatory cytokine, such as TNF-α, and adhesion molecules, such as VCAM-1 and ICAM-1. NF-κB has long been considered to play both proinflammatory and antiapoptotic roles, but its functions are context-dependent and therefore require cautious interpretation [122]. Specifically, activating NF-κB in non-parenchymal cells such as stellate cells, Kupffer cells, and sinusoidal endothelial cells, in general, increase inflammation and fibrosis, while suppressing NF-κB activation in parenchymal cells lead to hepatocarcinogenesis [121]. In light of the complexity of the NF-κB system and its cell-type specific functions, further research to elucidate its association with the hepatoprotective effect of *S. miltiorrhiza* extract might be needed.

### 7.4. S. miltiorrhiza Acts on Multiple Targets and Exhibits a Neuroprotective Effect on Several Nervous System Diseases

Regarding nervous system diseases, there has been ongoing research into how *S. miltiorrhiza* extract can exert neuroprotective effects against multiple diseases including Alzheimer’s disease (AD), ischemic stroke, and other neurological diseases. Since the advent of cholinesterase inhibitors (ChEIs) and *N*-methyl-d-aspartate (NMDA) receptor antagonist, the two drug classes currently available for AD treatment, extensive research has been conducted to find out novel therapeutic targets to treat AD, including butyrylcholinesterase (BuChE), β-secretase (BACE1), tau protein and related enzymes (e.g., GSK-3β), and oxidative stress [123]. In keeping with these trends, several recent studies included in our review investigated different mechanisms regarding the neuroprotective effect of *S. miltiorrhiza* extract in Aβ_25-35_-induced mouse or cell model of AD. For instance, Yu et al. demonstrated that treatment with *S. miltiorrhiza* extract at doses of 0.01, 0.1, 0.2 mg/mL for 2 h inhibited ROS generation in SH-SY5Y cells [92]. In addition, Paudel et al. reported on the inhibitory effect of *S. miltiorrhiza* extract on GSK-3β [93], while Ozarowski et al. examined the inhibitory effect of *S. miltiorrhiza* extract administration at a dose of 200 mg/kg for 28 days on AChE as well as BuChE and BACE1 in the rat brain cortex [94]. These multi-functional properties of *S. miltiorrhiza* extract suggest that it could act on multiple targets for the treatment of cognitive impairment and neurodegeneration. On the other hand, several other studies included in our review addressed the beneficial effect of *S. miltiorrhiza* extract on brain ischemic stroke, highlighting the antiplatelet [99], anti-inflammatory [98,100], and anti-apoptotic activities [97] of *S. miltiorrhiza* extract. Specifically, Fei et al. reported on the anti-platelet activity of *S. miltiorrhiza* extract in a mouse model of cerebral ischemic injury and suggested the involvement of PLC and PKC in the antiplatelet effect of *S. miltiorrhiza* [99]. According to Wang et al., the neuroprotective effect of *S. miltiorrhiza* was attributed to its anti-inflammatory properties, as evidenced by the reduction of the expression of RAGE, MMP-9, and other inflammatory factors (COX, TNF-α, ICAM-1), as well as its possible antioxidant activity evidenced by the activation of Nrf2/HO-1 signaling pathway [98]. Interestingly, Zhang et al. delved into the mechanism by which *S. miltiorrhiza* exerted a neuroprotective effect in a mouse model of spinal cord injury (SCI) [104]. *S. miltiorrhiza* extract administration at a dose of 12.5 g/kg for 8 days improved locomotor function and histopathological changes in mice and up-regulated NF-H, BDNF, CD11b protein expression. They further explored its possible mechanism using metabolomics, identifying 51 SCI-specific metabolites and suggesting 6 metabolic pathways where the altered metabolites were significantly impacted by *S. miltiorrhiza* extract, including vitamin B6 metabolism, pentose and glucuronate interconversions, lysine degradation starch and sucrose metabolism, arachidonic acid metabolism, and steroid hormone biosynthesis. This kind of state-of-the-art approach to unravel the therapeutic mechanism of *S. miltiorrhiza* could contribute to the deeper understanding of its therapeutic potentials.

### 7.5. Limitaions and Strong Points of This Study

Due to the time constraints on our review, we used PubMed as the only source of documents, set a 5-year time limit to our search for literature, excluded articles that were written in a language other than English, and focused our review on studies on specific disease groups that have been relatively well recognized and understood. In addition, heterogeneity between experiments may have influenced the interpretation of the results. Despite these limitations, our review was able to provide an overview of the efficacy of *S. miltiorrhiza* extract in various kinds of diseases so that the reader can obtain a broad picture of its multifaceted therapeutic benefits. In addition, we reviewed *S. miltiorrhiza* extract in multiple dosage forms, offering insights into how *S. miltiorrhiza* extract can deliver its therapeutic effects in various forms. This review can be regarded as a guide to further investigation rather than a conclusive analysis.

## 8. Conclusions

This review summarized recent experimental findings on the therapeutic effects of *S. miltiorrhiza* on cancers, cardiovascular, liver, and nervous system diseases. *S. miltiorrhiza* extract has been demonstrated to exert anti-inflammatory, anti-fibrotic, antioxidative, anti-apoptotic or pro-apoptotic, and neuroprotective effects in cell or animal model of such diseases. Despite current efforts to unravel the mechanism implicated in the multiple therapeutic effects of *S. miltiorrhiza*, it remains a major challenge to elucidate its multi-faceted therapeutic potentials.

## Figures and Tables

**Table 1:** Cancer and *S. miltiorrhiza.*

Disease	Extract	Experimental Model	Dose; Duration	Efficacy	Mechanism	Reference
Breast cancer	*70% ethanol*	MCF-7	50 µg/mL; 24 h	Inhibition of breast cancer cell invasiveness	↓ MMP-9, p-ERK, p-JNK, p-p38, p-c-Jun	[30]
Hepatocellular carcinoma	*Astragalus and Salvia miltiorrhiza water/ethanol extract (71:1.85)*	(1) SD rat (2) TGF-β1-stimulated HepG2 (3) BALB/c xenograft mouse model	(1) 60, 120, 240 mg/kg; 28 days (2) 20, 40, 80 µg/mL; 12, 24 h (3) 310 mg/kg; 28 days	Inhibition of hepatocellular carcinoma progression	↑ Smad3C, miR-145 ↓ Smad3L, miR-21, p-ERK, p-JNK, p-p38	[31]
Hepatocellular carcinoma	*Astragalus and Salvia miltiorrhiza water/ethanol extract (71:1.85)*	(1) SD rat (2) HSCs, HepG2	(1) 60, 120, 240 mg/kg; 12, 16 weeks (2) 20, 40, 80 µg/mL; 24 h	Inhibition of hepatocellular carcinoma	(1) ↑ pSmad3C ↓ p-ERK, p-JNK, p-p38, pSmad3L, Smad4, Imp 7/8, PAI-1 (2) ↑ p38 ↓ p-ERK, p-JNK	[32]
Multiple myeloma and myeloid leukemia	*99.9% ethanol*	U266, U937	25, 50, 100, 200 µg/mL; 24 h	Induction of apoptosis	↑ miR-216b, p-ATF4, p-eIf2, p-PERK, ROS, CHOP, c-PARP, c-caspase-3 ↓ c-Jun	[33]
Non-small cell lung cancer (NSCLC)	*Methanol extract* *(CTN-compounds of tanshinone)*	(1) Glc-82 (2) BALB/c mice	(1) 20, 40 µg/mL; 24 h (2) 40 mg; 22 days	Induction of apoptosis	↑ p53, p21, c-caspase-3, -9, c-PARP1, PTEN, Bax ↓ Bcl-2, Bcl-xl, p-Akt	[34]
Non-small cell lung cancer (NSCLC)	*Oldenlandia diffusa, Salvia miltiorrhiza 50% EtOH extract (5:2)*	(1) A549, H460 (2) HUVECs (3) H460 xenograft model	(1,2) PR 2.5 µg/mL + OS 180 µg/mL; 24 h (3) PR 125 µg/kg + OS 20 mg/kg; 18 days	Antiangiogenic and apoptotic effects	↑ c-caspase-3 ↓ p-STAT3, pro-PARP, Bcl-2, cyclin E, cyclin A, CDK2, E2F1, p-ERK, p-Akt, COX-2, SOCS-1, p-Src, VEGF, p-VEGFR2	[35]
Oral cancer	*Double-distilled water, 95% ethanol or 1:1 water/ethanol*	(1) HSC-3, OC-2 (2) BALB/cNU mice	(1) 10, 25, 50 µg/mL; 48, 72 h (2) 50, 100 mg/kg; 34 days	Inhibition of oral squamous carcinoma cell proliferation	↑ c-caspase-3 ↓ XIAP, survivin	[36]
Oral cancer	*95% ethanol*	(1) SAS, SCC25, Oec-ml (2) KB, KB7D, KB tax, KB100, KB Vin, KB Vin 10 (3) SAS xenograft animal model	(1) 0.625, 1.25, 2.5, 5, 10, 20, 30 µg/mL; 24 h (2) 2.5, 5, 10, 20, 40, 80 µg/mL; 24 h (3) 10 mg/kg; 32 days	Inhibition of proliferation of oral cancer cell	↑ c-caspase-3 ↓ XIAP	[37]
Prostate cancer	*Acetonitrile*	(1) PC-3 (2) PC-3 xenograft mouse model	(1) 20 µg/mL; 24, 48, 72 h (2) 100 mg/kg; 6 weeks	Inhibitory effect on the growth of prostate cancer cell	↑ ROS, c-caspase-3, -9, c-PARP, p21 ↓ Bcl-2, CDK2, CDK4, cyclin D1	[38]
Various cancers	*100% ethanol or 100% acetone*	AGS, A549, HCT116, LNCaP, MCF7	5, 10, 20, 40 µg/mL; 24 h	Inhibitory effect on the growth of cancer cells		[39]
Multidrug-resistant cancer	*Dichloromethane-methanol (1:1)*	CCRF-CEM	(1) 3, 10, 30 µg/mL; 1 h (2) 5, 10, 20, 40 µg/mL; N/A (3) 20 µg/mL; 24, 2 h	Cytotoxicity towards multidrug-resistant cancer cells	↑ ROS, p-JNK, p-ERK1/2, p-p38, c-caspase-3, -7, -9, c-PARP ↓ p65 translocation	[40]

MMP-9, matrix metalloproteinase-9; MAPK, mitogen-activated protein kinase; AP-1, activator protein-1; SD, Sprague Dawley; TGF-β, transforming growth factor-β; miR-145, microRNA-145; miR-21, microRNA-21; p-ERK, phospho-extracellular-signal-regulated-kinase; p-JNK, phospho-c-Jun N terminal kinase; p-p38, phosho-p38; HSCs, hepatic stellate cells; Imp, importins; PAI-1, plasminogen activator inhibitor 1; p-ATF4, phospho-activating transcription factor 4; p-eIF2, phospho-eukaryotic initiation factor 2; p-PERK, phospho-protein kinase RNA-like endoplasmic reticulum kinase; CHOP, CCAAT-enhancer-binding protein homologous protein; c-PARP, cleaved poly ADP-ribose polymerase; c-caspase-3, cleaved caspase-3; CTN, compounds of tanshinone; PTEN, phosphatase and tensin homolog deleted on chromosome ten; Bax, Bcl-2 associated X-protein; Bcl-2, B-cell lymphoma 2; Bcl-xl, B-cell lymphoma-extra large; p-Akt, phospho-Akt (protein kinase B); HUVEC, human umbilical vein endothelial cell; PR, tetraarsenic hexoxide; OS, Olendlandia diffusa and Salvia miltiorrhiza extract; p-STAT3, phospho-signal transducer and activator of transcription 3 (Tyr705); VEGF, vascular endothelial growth factor; CDK, cyclin-dependent kinase; XIAP, X-linked inhibitor of apoptosis protein; ROS, reactive oxygen species.

**Table 2:** Cardiovascular diseases and *S. miltiorrhiza.*

Disease	Extract	Experimental Model	Dose; Duration	Efficacy	Mechanism	Reference
Coronary heart disease (CHD)	*Extractant unmentioned (Danshen pills)*	Patients	810 mg/day; 3 months	Reduction of the CHD risk	↑ HDL-C, ApoA, ApoB, ApoE, TBil, IBil ↓ TG, TC, LDL-C, Lp(a), GGT, DBil, UA, Hcy	[48]
Iron-mediated myocardial fibrosis	*Water*	Kunming mice	3, 6 g/kg; 7 weeks	Protective effect on cardiac fibrosis induced by chronic iron overload	↑ SOD ↓ TGF- β_1_, MMP-9, COL I, COL III	[49]
Myocardial infarction (MI)	*Extractant unmentioned* *(Danshen injection)*	BALA/c mice	3, 6 g/kg; 4 weeks	Beneficial effect on cardiac angiogenesis and cardiac function	↑ HIF1α, VEGFA	[50]
Myocardial infarction (MI)	*Water*	SD rats	1.5 mL/kg; 14 days	Anti-inflammatory and anti-cardiac remodeling effects	↑ Bcl-2/Bax ↓ MMP-2, MMP-9, iNOS, MPO	[51]
Myocardial infarction (MI)	*Salvia miltiorrhiza and Carthamus tinctorius extract (ratio unmentioned)*	Wild-type C57BL/6 mice	3 µL/g; 3 weeks	Inhibition of inflammation and fibrosis	↑ IL-10 ↓ H3K4me3, H3K36me3, IL-1β, TNF, IL-6, COL I, COL III, α-SMA, Col1a1, Col3a1, Acta2	[52]
Myocardial infarction (MI)	*Salvia miltiorrhiza Bunge and Astragalus mongholicus extract (1:1)*	SD rats	20 mg/kg/day; 8 weeks	Inhibition of myocardial fibrosis and ventricular remodeling	↓ PKD1	[53]
Myocardial ischemia	*Salvia miltiorrhiza Bunge and Panax notoginseng water extract (1:1)*	SD rats	1.2 mg/kg; 28 days	Regulatory effect on lipid metabolism disorder induced by myocardial ischemia	↑ ApoA-I, FATP, CPTI, PPARα, RXR ↓ TG, LDL, Apo-B, HMGCR, NR2C2	[54]
Post-MI complications	*Salvia miltiorrhiza Bunge and Panax notoginseng powdered water extract (1:1)*	SD rats	0.6, 0.9, 1.2 g/kg; 4 weeks	Inhibition of infarct border zone remodeling and ventricular arrhythmias	↑ Cx43 ↓ TGF-β_1_, COL I, COL III, α-SMA, p-Smad3, BNP, MCP-1	[55]
Ischemia-reperfusion injury, cardiac hypertrophy, hypertension, and inflammation	*75% ethanol*	sEH, 8,9-EET	IC_50_: 86.5 µg/mL	Cardiovascular protective and anti-inflammatory effects	↓ sEH activity, 8,9-DHET	[56]
Myocardial ischemia/reperfusion (I/R) and hypoxia/reoxygenation injuries	*Salvia miltiorrhiza and ligustrazine injection (1:50)*	SD rats	6.8, 20.4, 61.2 mg/kg/day; 3 days	Alleviation of I/R injury in cardiomyocytes and inhibition of apoptosis	↑ Bcl-2/Bax, p-Akt, p-eNOS, SOD ↓ caspase-3, MDA	[57]

HDL-C, high-density lipoprotein cholesterol; ApoA, apolipoprotein A; ApoB, apolipoprotein B; ApoE, apolipoprotein E; TBil, total bilirubin; IBil, indirect bilirubin; TG, triglycerides; TC, total cholesterol; LDL-C, low-density lipoprotein cholesterol; Lp(a), lipoprotein (a); GGT, gamma-glutamyl transpeptidase; DBil, direct bilirubin; UA, uric acid; Hcy, homocysteine; SOD, superoxide dismutase; Hyp, hydroxyproline; TGF-β1, transforming growth factor-β1; MDA, malondialdehyde; MMP, matrix metalloproteinase; COL I, type I collagen; COL III, type III collagen; HIF1α, hypoxia-inducible factor 1α; SD, Sprague Dawley; VEGFA, vascular endothelial growth factor A; Bcl-2, B-cell lymphoma 2; Bax, Bcl-2 associated X protein; iNOS, inducible nitric oxide synthase; MPO, myeloperoxidase; IL-10, interleukin-10; H3K4me3, H3K4 trimethylation; H3K36me3, H3K36 trimethylation; IL-1β, interleukin-1β; TNF, tumor necrosis factor; IL-6, interleukin-6; α-SMA, α-smooth muscle actin; Col1a1, collagen type I alpha 1; Col3a1, collagen type 3 alpha 1; Acta2, actin alpha 2; PKD1, protein kinase D1 protein; FATP, fatty acids transport protein; CPTI, carnitine palmitoyltransferase I; PPARα, peroxisome proliferator-activated receptor–α; RXR, retinoid X receptor; HMGCR, 3-hydroxy-3-methylglutaryl-coenzyme A reductase; NR2C2, nuclear receptor subfamily 2 group C member 2; Cx43, connexin 43; p-Smad3, phospho-Smad3; BNP, brain natriuretic peptide; MCP-1, monocyte chemoattractant protein-1; sEH, soluble epoxide hydrolase; 8,9-EET, 8,9-epoxyeicosatrienoic acid; 8,9-DHET, 8,9-dihydroxyeicosatrienoic acids; p-Akt; phospho-Akt (protein kinase B); p-eNOS, phospho-endothelial nitric oxide synthase.

**Table 3:** Liver diseases and *S. miltiorrhiza.*

Disease	Extract	Experimental Model	Dose; Duration	Efficacy	Mechanism	Reference
Alcoholic liver disease (ALD)	*Extractant unmentioned* *(Danshen injection)*	(1) C57BL/6 mice (2) HepG2 (3) NCTC1469	(1) 3 g/kg; 4 weeks (2) 100, 200 µg/mL; 2 h (3) 150 µg/mL; 2 h	Hepatoprotective effect against ALD	↑ PPARα, CPT-1, CPT-2 ↓ 4-HNE	[65]
Alcoholic liver fibrosis (ALF)	*Pueraria lobata, Salvia miltiorrhiza, Schisandra chinensis, Silybum marianum extract (8:5:4:3)*	SD rats	0.333, 0.667, 1 g/kg; 30 days	Anti-fibrotic effect	↑ MMP-13, Smad7 ↓ TIMP-1, TGF-β_1_, p-Smad2, p-Smad3	[66]
APAP-induced hepatotoxicity	*Water*	(1) Primary SD rat hepatocytes (2) SD rat liver microsomes	(1) 0.25, 1 mg/mL; 24 h (2) 0.25, 1 mg/mL; 24 h	Antioxidant and anti-hepatotoxic effects	↑ GSH/GSSG ratio ↓ CYP2E1	[68]
Hepatic sinusoidal obstruction syndrome (HSOS)	*Extractant unmentioned*	KM mice	100, 200 mg/kg; N/A	Hepatoprotective effect on *Gynura segetum-*induced HSOS	↓ TNF-α, VCAM-1, ICAM-1, NF-κB p65	[69]
LPS-induced liver injury	* Salvia miltiorrhiza, Carthamus tinctorius extract (5:2) *	C57BL/6J mice	3 g/kg; 30 min	Anti-inflammatory, anti-oxidative, and anti-apoptotic effects	↑ Bcl-2 ↓ TNF-α, IL-6, p-NF-κB p65, p-IκBα, Bax	[70]
Liver fibrosis	*Ethanol*	SD rats	1, 2.5 mg/kg; 12 weeks	Anti-fibrotic effect against TAA-induced liver fibrosis	↓ COL I (α), TIMP-1, α-SMA	[71]
Liver fibrosis	*90% ethanol*	(1) C57BL/6 mice (2) NK cells (3) JS-1, NK cells (1:50)	(1) 1.5, 3.0 g/kg; 4 weeks (2, 3) 50 µg/mL; 16 h	Anti-fibrotic effect against CCl_4_-induced liver fibrosis	(1, 2) ↑ NKG2D, NKp46, IFN-γ (3) ↑ RAE-1ε, ↓ α-SMA	[72]
Non-alcoholic steatohepatitis (NASH)	* 70% ethanol *	(1) C57BL/6j mice (2, 3) LX-2	(1) 0.5, 1 mg/kg; 4, 6 weeks (2) 0.1, 0.5, 1 µg/mL; 24 h (3) 0.1, 1, 10, 100 µg/mL; 30 min	Anti-inflammatory, anti-fibrotic, and antioxidant effects	↓ TNF-α, TGF-β_1_, IL-1β, α-SMA, COL I, MMP-2, MMP-9, ROS	[73]
Non-alcoholic steatohepatitis (NASH)	*Artemisia iwayomogi, Amomum xanthioides, Salvia miltiorrhiza* *water extract* *(CGplus)*	C57/BL6J mice	50, 100, 200 mg/kg; 5 days	Protection against the development of NASH	↑ (p)-AMPK, ACADL, IL-10 ↓ TNF-α, IL-1β, IL-6, HMGCR, p-SREBP-1	[74]
Non-alcoholic fatty liver disease (NAFLD)	*Atrctylodes macrocephaly, Salvia miltiorrhiza, Radix Paeonia Alba, Rhizoma Alismatis, Fructus Schisandrae Chinensis powdered extract* *(JPHX formula)*	Wistar rat	0.60, 1.21, 2.42 g/kg; 8 weeks	Hepatoprotective effect against NAFLD	↓ TG, TC, TNF-α, COL I, MMP-9, p-JNK	[75]

PPAR-α, peroxisome proliferator-activated receptor alpha; CPT-1, carnitine palmitoyltransferase 1; CPT-2, carnitine palmitoyltransferase 2; 4-HNE, 4-hydroxynonenal; MMP-13, matrix metalloproteinase-13; TIMP-1, tissue inhibitor of metalloproteinase-1; SD, Sprague Dawley; ALF, alcohol liver fibrosis; APAP, acetaminophen; CYP2E1, cytochrome P450 2E1; KM, kunming mice; GSH/GSSG ratio, glutathione/glutathione disulfide ratio; VCAM-1, vascular cell adhesion molecule 1; ICAM-1, intercellular adhesion molecule-1; Bcl-2, B-cell lymphoma 2; TNF- α, tumor necrosis factor- α; IL-6, interleukin -6; MDA, malondialdehyde; p-NF-κB, phospho-nuclear factor kappa-light-chain-enhancer of activated B cells; p-IκBα, phospho-inhibitor of nuclear factor kappa B α; Bax, Bcl-2 associated X-protein; TAA, thioacetamide; TIMP-1, tissue inhibitor of metalloproteinases-1; α-SMA, alpha-smooth muscle actin; CCl_4_, carbon tetrachloride; NKG2D, natural killer group 2D; NKp46, natural killer p46; IFN-γ, interferon gamma; RAE-1ε, retinoic acid early-inducible protein 1 ε; TGF-β_1_, transforming growth factor beta 1; IL-1β, interleukin 1 beta; CRP, c-reactive protein; COL I, type I collagen; MMP-2, matrix metalloproteinase-2; MMP-9, matrix metalloproteinase-9; ROS, reactive oxygen species; (p)-AMPK, phospho-AMP-activated protein kinase; ACADL, acyl-CoA dehydrogenase long chain; HMGCR, 3-hydroxy-3-methyl-glutaryl-coenzyme A reductase; p-SREBP-1, phospho-sterol regulatory element-binding protein 1; TG, triglycerides; TC, total cholesterol; p-JNK, phospho-c-Jun N-terminal kinase.

**Table 4:** Nervous system diseases and *S. miltiorrhiza*.

Disease	Extract	Experimental Model	Dose; Duration	Efficacy	Mechanism	Reference
Alzheimer’s disease (AD)	*Water*	SH-SY5Y cells	0.01, 0.1, 0.2 mg/mL; 2 h	Neuroprotection against Aβ_25-35_-induced neurotoxicity	↓ ROS, Bax/Bcl-2, cytochrome c, caspase-3	[92]
Alzheimer’s disease (AD)	*Water*	Human recombinant GSK-3β	IC_50_: 7.77±1.38 μg/mL	Inhibition of AD	↓ GSK-3β	[93]
Alzheimer’s disease (AD)	*50% ethanol*	Wistar rats	200 mg/kg; 28 days	Improvement of long-term memory of rats	↓ AChE, BuChE, BACE1	[94]
Alzheimer’s disease (AD)	*Salvia miltiorrhiza, Panax Notoginseng, Borneol extract (450:141:8)*	Kunming mice	0.405, 0.81 g/kg; 7 days	Neuroprotective, anti-inflammatory, neurotrophic effects on learning and memory in Aβ_25-35_-induced mice	↑ ChAT, BDNF, RACK1 ↓ IL-6, TNF-α	[95]
Alzheimer’s disease (AD)	*Salvia miltiorrhiza, Panax Notoginseng, Borneol ethanol extract (450:141:8)*	SD rats	520 mg/kg; 14 days	Improvement of spatial learning and memory in Aβ_25-35_-induced rat model of AD	↑ IDE ↓ APP, PS1	[96]
Brain Ischemic Stroke	*Water*	(1) MSCs (2) SD rats	(1) 10 µg/mL (2) 50 mg/kg; 2 weeks	Anti-apoptosis and improvement of cell survival	↑ Bcl-2, p-Akt, p-ERK ↓ Bax, caspase-3	[97]
Cerebral Ischemia (Acute)	*80% ethanol*	Wistar rats	5.25, 10.5, 21 mg/kg; 15 days	Neuroprotective effect against cerebral ischemic injury	↑ HO-1, HQO-1, Nrf-2 ↓ RAGE, MMP-9, COX-2, TNF-α, ICAM-1	[98]
Cerebral Ischemia (Permanent)	*Supercritical CO_2_ and 95% ethanol*	SD rats	(1)15, 7.5, 3.75 mg/kg/day; 3 days (2) 0.44, 4.4, 44 mg/L; 10 min	Attenuation of cerebral ischemic injury through inhibitory effects on thrombosis formation and platelet aggregation in rats	↓ TXA_2,_ p-PLCβ3, p-PKC	[99]
Cerebral Ischemia	*Salvia miltiorrhiza, Ligusticum chuanxiong, Carthamus tinctorius water extract* *—* *Ratio unmentioned*	Kunming mice	(1) 20 g/kg; 5 days (2) 20 g/kg; 28 days	Recovery of cognitive impairment and Neuroprotection against cerebral ischemic injury	↑ Bcl-2, BDNF ↓ IL-1β, IL-6, TNF-α, Bax	[100]
Dementia	*Extractant unmentioned*	SD rats	5 mL/kg/day; 4 weeks	Improvement of learning and memory abilities in streptozotocin-induced diabetic rats	↑ MKP-1	[101]
Dementia, Vascular	*Water*	Wister rats	200 mg/kg/day; 22 days	Protection against damage to the white matter and hippocampus after bilateral common carotid artery occlusion	↑ MBP ↓ TNF-α, IL-1β, IL-6, TLR4, MyD88	[102]
Ethanol-induced Amnesia	*70% ethanol*	CD-1 mice	(1) 200 mg/kg; 30 min (2) 10, 100 µg/mL; 20 min	Blockage of ethanol-induced synaptic dysfunction	↑ LTP, NMDAR-dependent fEPSP	[103]
Spinal cord injury (SCI)	*75% ethanol*	SD rats	12.5 g/kg; 8 days	Beneficial effects on the recovery of locomotor function after SCI	↑ NF-H, BDNF, CD11b	[104]
Temporal Lobe Epilepsy (TLE)	*Extractant unmentioned* *Salvia miltiorrhiza Bunge, Panax notoginseng, Borneol—Ratio unmentioned*	SD rats	85 mg/kg; 90 days	Neuroprotection on a kainic acid-induced TLE and cognitive impairment in rats	↑ GDNF, Bcl-2/Bax	[105]

ROS, reactive oxygen species; Bax, Bcl-2 associated X protein; Bcl-2, B-cell lymphoma 2; GSK-3β, glycogen synthase kinase-3β; AchE, acetylcholinesterase; BuChE, butyrylcholinesterase; BACE1, β-secretase; ChAT, choline acetyltransferase; BDNF, brain derived neurotrophic factor; RACK 1, receptor of activated protein kinase C1; IL-6, interleukin-6; TNF- α, tumor necrosis factor- α; IDE, insulin-degrading enzyme; APP, amyloid precursor protein; PS1, presenilin-1; MSCs, mesenchymal stem cells; p-Akt, phospho-Akt (protein kinase B); p-ERK, phospho-extracellular signal-regulated kinase; HO-1, heme oxygenase-1; HQO-1, NAD(P)H quinine oxidoreductase; Nrf2, nuclear factor erythroid 2-related factor 2; RAGE, receptor for advanced glycation endproducts; MMP-9, matrix metalloproteinase-9; COX-2, cyclooxygenase-2; ICAM- 1, intercellular adhesion molecule-1; TXA2, thromboxane A2; pPLCβ3, phospho-phospholipase Cβ3; p-PKC, phospho-protein kinase C; IL-1β, interleukin-1β; MKP-1, mitogen-activated protein kinase-1; MBP, myelin basic protein; TLR4, toll-like receptor 4; MyD88, myeloid differentiation primary response 88; LTP, long-term potentiation; NMDAR, N-methyl-d-aspartate receptor; fEPSP, field excitatory postsynaptic potential; NF-H, neurofilament 200; CD11b, cluster of differentiation molecule 11B; GDNF, glial cell line-derived neurotrophic factor.

## Glossary

4-HNE	4-hydroxynonenal
8	9-DHET
8	9-dihydroxyeicosatrienoic acids
8	9-EET
8	9-epoxyeicosatrienoic acid
ACADL	acyl-CoA dehydrogenase long chain
AchE	acetylcholinesterase
Acta2	actin alpha 2
Akt	protein kinase B
ALF	alcohol liver fibrosis
AMPK	AMP-activated protein kinase
AP-1	activator protein-1
APAP	acetaminophen
Apo	apolipoprotein
APP	amyloid precursor protein
ATF4	activating transcription factor 4
BACE1	β-secretase
Bax	Bcl-2 associated X-protein
Bad	Bcl-2 associated agonist of cell death
Bcl-2	B-cell lymphoma 2
Bcl-xl	B-cell lymphoma-extra large
BDNF	brain derived neurotrophic factor
BNP	brain natriuretic peptide
BuChE	butyrylcholinesterase
c-caspase-3	cleaved caspase-3
c-PARP	cleaved poly ADP-ribose polymerase
CCl4	carbon tetrachloride
CD11b	cluster of differentiation molecule 11B
CDK	cyclin-dependent kinase
ChAT	choline acetyltransferase
CHOP	CCAAT-enhancer-binding protein homologous protein
COL I	type I collagen
COL III	type III collagen
Col1a1	collagen type I alpha 1
Col3a1	collagen type 3 alpha 1
COX-2	cyclooxygenase-2
CPTI	carnitine palmitoyltransferase I
CRP	c-reactive protein
CTN	compounds of tanshinone
Cx43	connexin 43
CYP2E1	cytochrome P450 2E1
DBil	direct bilirubin
eIF2	eukaryotic initiation factor 2
eNOS	endothelial nitric oxide synthase.
ERK	extracellular-signal-regulated-kinase
ERK	extracellular signal-regulated kinase
FATP	fatty acids transport protein
fEPSP	field excitatory postsynaptic potential
GDNF	glial cell line-derived neurotrophic factor
GGT	gamma-glutamyl transpeptidase
GSH/GSSG ratio	glutathione/glutathione disulfide ratio
GSK-3β	glycogen synthase kinase-3β
H3K36me3	H3K36 trimethylation
H3K4me3	H3K4 trimethylation
Hcy	homocysteine
HDL-C	high-density lipoprotein cholesterol
HIF1α	hypoxia-inducible factor 1α
HMGCR	3-hydroxy-3-methylglutaryl-coenzyme A reductase
HO-1	heme oxygenase-1
HQO-1	NAD(P)H quinine oxidoreductase
HSCs	hepatic stellate cells
HUVEC	human umbilical vein endothelial cell
Hyp	hydroxyproline
IBil	indirect bilirubin
ICAM-1	intercellular adhesion molecule-1
IDE	insulin-degrading enzyme
IFN-γ	interferon gamma
IL-10	interleukin-10
IL-1β	interleukin 1 beta
IL-6	interleukin-6
Imp	importins
iNOS	inducible nitric oxide synthase
IκBα	inhibitor of nuclear factor kappa B α
JNK	c-Jun N terminal kinase
KM	kunming Mice
LDL-C	low-density lipoprotein cholesterol
Lp(a)	lipoprotein (a)
LTP	Long-term potentiation
MAPK	mitogen-activated protein kinase
MBP	Myelin basic protein
MCP-1	monocyte chemoattractant protein-1
MDA	malondialdehyde
miR-145	microRNA-145
miR-21	microRNA-21
MKP-1	mitogen-activated protein kinase-1
MMP	matrix metalloproteinase
MPO	myeloperoxidase
MSCs	mesenchymal stem cells
MyD88	myeloid differentiation primary response 88
NF-H	neurofilament 200
NF-κB	nuclear factor kappa-light-chain-enhancer of activated B cells
NKG2D	natural killer group 2D
NKp46	natural killer p46
NMDAR	N-methyl-d-aspartate receptor
NR2C2	nuclear receptor subfamily 2 group C member 2
Nrf2	nuclear factor erythroid 2-related factor 2
NSCLC	non-small cell lung cancer
OS	Olendlandia diffusa and Salvia miltiorrhiza extract
PAI-1	plasminogen activator inhibitor 1
PERK	protein kinase RNA-like endoplasmic reticulum kinase
PKC	protein kinase C
PKD1	protein kinase D1 protein
PPAR-α	peroxisome proliferator-activated receptor—alpha
pPLCβ3	phospho-phospholipase Cβ3
PR	tetraarsenic hexoxide
PS1	Presenilin-1
PTEN	phosphatase and tensin homolog deleted on chromosome ten
RACK 1	receptor of activated protein kinase C1
RAE-1ε	retinoic acid early-inducible protein 1 ε
RAGE	receptor for advanced glycation endproducts
ROS	reactive oxygen species.
RXR	retinoid X receptor
SD	Sprague Dawley
sEH	soluble epoxide hydrolase
SOD	superoxide dismutase
SREBP-1	sterol regulatory element-binding protein 1
STAT3	signal transducer and activator of transcription 3 (Tyr705)
TAA	thioacetamide
TBil	total bilirubin
TC	total cholesterol
TG	triglycerides
TGF-β1	transforming growth factor beta 1
TIMP-1	tissue inhibitor of metalloproteinase-1
TLR4	toll-like receptor 4
TNF	tumor necrosis factor
TXA2	thromboxane A2
UA	uric acid
VCAM-1	vascular cell adhesion molecule 1
VEGFA	vascular endothelial growth factor A
XIAP	X-linked inhibitor of apoptosis protein
α-SMA	α-smooth muscle actin
